# The Transfer of the Ferredoxin Gene From the Chloroplast to the Nuclear Genome Is Ancient Within the Paraphyletic Genus *Thalassiosira*

**DOI:** 10.3389/fmicb.2020.523689

**Published:** 2020-10-02

**Authors:** Alexandra-Sophie Roy, Christian Woehle, Julie LaRoche

**Affiliations:** ^1^Genomic Microbiology, Institute of General Microbiology, Kiel University, Kiel, Germany; ^2^Max Planck-Genome-Centre Cologne, Max Planck Institute for Plant Breeding Research, Cologne, Germany; ^3^Department of Biology, Dalhousie University, Halifax, NS, Canada

**Keywords:** ferredoxin, gene transfer, *Thalassiosira* genus, *T. pseudonana*, *T. weissflogii*, PETF, *T. oceanica*, phylogenetics

## Abstract

Ferredoxins are iron–sulfur proteins essential for a wide range of organisms because they are an electron transfer mediator involved in multiple metabolic pathways. In phytoplankton, these proteins are active in the mature chloroplasts, but the *petF* gene, encoding for ferredoxin, has been found either to be in the chloroplast genome or transferred to the nuclear genome as observed in the green algae and higher plant lineage. We experimentally determined the location of the *petF* gene in 12 strains of *Thalassiosira* covering three species using DNA sequencing and qPCR assays. The results showed that *petF* gene is located in the nuclear genome of all confirmed *Thalassiosira oceanica* strains (CCMP0999, 1001, 1005, and 1006) tested. In contrast, all *Thalassiosira pseudonana* (CCMP1012, 1013, 1014, and 1335) and *Thalassiosira weissflogii* (CCMP1010, 1049, and 1052) strains studied retained the gene in the chloroplast genome, as generally observed for Bacillariophyceae. Our evolutionary analyses further extend the dataset on the localization of the *petF* gene in the Thalassiosirales. The realization that the *petF* gene is nuclear-encoded in the *Skeletonema* genus allowed us to trace the *petF* gene transfer back to a single event that occurred within the paraphyletic genus *Thalassiosira*. Phylogenetic analyses revealed the need to reassess the taxonomic assignment of the *Thalassiosira* strain CCMP1616, since the genes used in our study did not cluster within the *T*. *oceanica* lineage. Our results suggest that this strains’ diversification occurred prior to the ferredoxin gene transfer event. The functional transfer of *petF* genes provides insight into the evolutionary processes leading to chloroplast genome reduction and suggests ecological adaptation as a driving force for such chloroplast to nuclear gene transfer.

## Introduction

Diatoms are widely recognized as some of the most ecologically successful eukaryotes on the planet (Armbrust, 2009), accounting for approximately 40% of the global net primary production (Nelson et al., 1995; Field et al., 1998). Although diatoms are widely investigated, the reason for their immense success is still not fully understood. The ecological success of diatoms has often been attributed to their functional plasticity allowing them to dominate in very diverse environments, from nutrient-rich coastal areas to Fe-depleted, high-nutrient, low-chlorophyll (HNLC) oceanic regions (Strzepek and Harrison, 2004). Iron (Fe) is known to limit the growth of diatoms in large areas of the oceans (Moore et al., 2004; Boyd et al., 2007), notably in (HNLC) regions where *in situ* Fe addition experiments have triggered large diatom blooms (Chisholm et al., 2001; Boyd et al., 2007). Although genetic adaptation can manifest itself on various levels, part of the adaptation mechanism in *Thalassiosira oceanica* (a diatom extremely tolerant to Fe-limitation) is related to the tight regulation of the stoichiometric composition of the components of the photosystem I (PSI) and II (PSII) and the intermediate electron carrier proteins (Strzepek and Harrison, 2004). Ferredoxin, as the first electron acceptor and an Fe-containing enzyme, will therefore play an important role in regulating the redox poise of the chloroplast electron transfer chain (Hanke and Mulo, 2013). In addition to the plasticity in *T. oceanica*’s photosynthetic architecture allowing a reduction in the Fe-rich PSI relative to PSII, replacement of cytochrome C6 with plastocyanin, a protein that does not contain Fe (Peers and Price, 2006), allows for further reduction in the cellular Fe quota. Similarly, the replacement of the Fe-containing protein PetF by flavodoxin (Fld1A) was observed in many diatoms in Fe-limited growth conditions (LaRoche et al., 1995; McKay et al., 1997).

While the evolutionary origin of plastid in photosynthetic eukaryotes has been firmly attributed to primary endosymbiosis of a cyanobacterium (Martin et al., 2002; Kleine et al., 2009), the complex plastids of diatoms and many other microalgae result from the secondary endosymbiosis of eukaryotic red algae harboring functional plastids (Archibald, 2009; Zimorski et al., 2014). Massive transfer of genetic content from the ancestral plastid genome to the host genome (termed endosymbiotic gene transfer) characterized the evolution following the endosymbiosis event resulting in major genomic modifications of both the nuclear and plastid genomes. In fact, the photosynthetic apparatus of diatoms involves more than 700 genes coordinated by the plastid and nuclear genomes. However, modern chloroplast genomes host only about 200 of these genes (Kleffmann et al., 2004). The remaining photosynthetic proteins are encoded in the nuclear genome, synthesized in the cytoplasm and imported in the plastid by complex import systems that have co-evolved with N-terminal targeting sequences (Garg and Gould, 2016). The coordinated regulation of chloroplast and nuclear encoded genes in variable environmental conditions is essential to ensure photosynthetic function. Although transfer events that take place on an evolutionary timescale are generally studied by a comparative genomic approach, experimental studies with transgenic plants have demonstrated that the frequency of gene transfer from the chloroplast to the nuclear genome is higher than previously thought (Stegemann et al., 2003). In diatoms, plastid genome is subject to frequent internal rearrangements (Oudot-Le Secq et al., 2007) that promote the transfer of organelle-encoded genes to the nuclear genome (Martin et al., 2002; Richly and Leister, 2004; Bock and Timmis, 2008; Sheppard and Timmis, 2009; Sabir et al., 2014). Further, the multiple endosymbiotic events together with special features of centromeres in diatoms may have provided an ecologically driven mechanism for remodelling of the chloroplast and nuclear genomes (Diner et al., 2017). In *T. oceanica* (Husdedt) Hasle & Heimdal CCMP1005 (Sunda and Huntsman, 1995; Strzepek and Harrison, 2004), a species with a true haploid nuclear genome (Lommer et al., 2012), the *petF* gene encoding for the ferredoxin protein PetF was functionally transferred from the chloroplast to the nuclear genome (Lommer et al., 2010). This is unusual because the *petF* gene is commonly found in the chloroplast genome of most diatoms including the closely related coastal species *Thalassiosira pseudonana* and *Thalassiosira weissflogii* (Grunow) G. Fryxell & Hasle (Gueneau et al., 1998; Lommer et al., 2012). The fact that *T. oceanica* maintains a higher growth rate under Fe limitation than *T. pseudonana* and *T. weissflogii* (Sunda et al., 1991; Maldonado and Price, 1996; Strzepek and Harrison, 2004) is interesting in regard to the *petF* gene transfer. Furthermore, it was established that the *petJ* gene, encoding for cytochrome C6, was transferred from the chloroplast genome to the nuclear genome in diatoms and that this transfer happened early in the evolution of diatoms, dating back to the separation between *Leptocylindrus* and all other diatoms (Ruck et al., 2014). It is speculated that some gene modifications such as deletions, inversions duplication, or other forms of adaptation could be evolutionary responses to environmental stress. For example, the presence of both *fld1A* and *petF* protein-coding gene in the nuclear genome may have originated as a response to Fe stress and possibly gave some diatoms an ecological advantage through time. It was also previously hypothesized that the functional transfer of the *petF* gene may provide *T. oceanica* an additional advantage to tolerate Fe limitation (Lommer et al., 2010). Here, we used quantitative polymerase chain reaction (qPCR) to determine whether the *petF* gene transfer to the nuclear genome was generally detectable across all strains of *T. oceanica* in addition to CCMP1005, for which such a transfer has been confirmed (Lommer et al., 2010). We also investigated whether the *petF* transfer to the nuclear genome is scattered throughout other isolated strains of the *Thalassiosira* genus. A common gene transfer at the origin of multiple strains could indicate for a shared benefit for the evolution of *Thalassiosira* genus. We cultivated and screened five strains of *T. oceanica* (CCMP0999, 1001, 1005, 1006, and 1616), four of *T. pseudonana* (CCMP1012, 1013, 1014, and 1335), and three of *T. weissflogii* (CCMP1010, 1049, and 1052) to determine the localization of the *petF* gene. The results established that the *petF* gene transfer is unique to *T. oceanica*. The experimental approach was complemented by phylogenetic reconstructions including homologs obtained from publicly available databases to extend the number of potential transferred *petF* genes by considering additional species from Thalassiosirales.

## Materials and Methods

### Strains and Cultures

A total of 12 strains from three *Thalassiosira* species (Table 1) were obtained from the National Center for Marine Algae and Microbiota (NCMA formerly CCMP^1^) and were cultured in sterile f/2 artificial seawater medium (Guillard and Ryther, 1962; Kester et al., 1967; Guillard, 1975). Each strain was cultured in duplicate at its optimal temperature and light conditions (Table 1) and was harvested in late exponential phase reached within 7 to 15 days. Cells were collected by filtration on polycarbonate filters (Durapore^®^ 47 mm, Millipore, Darmstadt, Germany) with pore sizes compatible to the algae’s size. Cells from each filter were resuspended in a small volume of sterile medium and centrifuged at 11,000 rpm for 10 min at 4°C to concentrate the cells in pellets, which were thereafter flash frozen in liquid nitrogen and stored at −80°C until DNA extraction was performed.

**TABLE 1:** Tested *Thalassiosira* strains environmental details (n/a = not available, Env. T = environmental temperature).

Species	Strain CCMP	Env. T (°C)	Origin	Environment	Taxonomic author(s)
*Thalassiosira Oceanica*	0999	22–26	Continental slope; North Atlantic	Warm core eddy	(Hustedt) Hasle & Heimdal
*T. oceanica*	1001	22–26	Continental slope; North Atlantic	n/a	(Husdedt) Hasle & Heimdal
*T. oceanica*	1005	22–26	Sargasso Sea	Oceanic	(Husdedt) Hasle & Heimdal
*T. oceanica*	1006	22–26	Sargasso Sea	Oceanic	(Husdedt) Hasle & Heimdal
*T. oceanica*	1616	22–26	Eastern Mediterranean Sea	n/a	(Husdedt) Hasle & Heimdal
*Thalassiosira pseudonana*	1012	11–16	Swan River Estuary, Perth, Australia	Estuarine	(Husdedt) Hasle & Heimdal
*T. pseudonana*	1013	11–16	Conwy, Gwynedd, Wales, United Kingdom	Estuarine	(Husdedt) Hasle & Heimdal
*T. pseudonana*	1014	11–16	North Pacific Gyre	n/a	(Husdedt) Hasle & Heimdal
*T. pseudonana*	1335	11–16	Long Island, NY, United States	Estuarine shallow bay	(Husdedt) Hasle & Heimdal
*Thalassiosira weissflogii*	1010	11–16	Stream, between Bermuda and New York	Oceanic	(Husdedt) Hasle & Heimdal
*T. weissflogii*	1049	11–16	Long Island Sound Sea, NY, United States	n/a	(Grunow) Fryxell & Hasle
*T. weissflogii*	1052	11–16	Skagerrak Sea, Oslo Fjord, Norway	n/a	(Grunow) Fryxell & Hasle

*Thalassiosira oceanica was cultured at 24.6°C, 70–90 μE, and 14/10-h light/dark cycles. T. pseudonana and T. weissflogii were cultured at 16°C, 100 μE, and 12/12-h light/dark cycles.*

### Nucleic Acid Extraction

Total nucleic acid was extracted using the DNeasy Plant mini kits from Qiagen^®^ (Qiagen, Hilden, Germany) following the standard manufacturer’s protocol, with minor modifications. Changes to the protocol included a 10-s sonication of the cell pellets following the addition of lysis buffer, the washing step with AW buffer was done twice, and the rinsing step with 500 μl of 96% ethanol was done thrice as recommended to increase yield. DNA was eluted in 250 μl of elution buffer and was finally sheared using a sterile hypodermic needle (30 gauge) attached to a sterile 1-ml syringe. DNA amounts were determined by micro-volume spectrophotometer nanodrop ND-1000 (PeqLab GmbH, Erlangen, Germany) as the mean of triplicate measurements, and quality of the DNA was tested using the automated electrophoresis system Experion^TM^ DNA-12K analysis kit from Bio-Rad Laboratories (Hercules, California, United States). All samples were kept at −80°C until further analysis.

### Quantitative Polymerase Chain Reaction

Gene transfer was assessed by comparing the threshold cycle (Ct) values of reference single-copy genes of known compartmental locations, either chloroplastic or nuclear, with the Ct values of the *petF* gene (chloroplastic, *petF*_CP_; or nuclear, *petF*_NUC_). The presence of multiple chloroplasts in *Thalassiosira* spp. results in multiple copies of chloroplast genomes; and because the chloroplast genome copies are naturally enriched in comparison with the nuclear genome, single-copy chloroplast-encoded genes are easily separated from single-copy nuclear-encoded genes. Single-copy genes located in the nuclear genome of eukaryotic diploid cells are present in two copies per cell, while genes located in the chloroplast genome have several copies.

Quantitative polymerase chain reaction was performed with SYBR green master mix on the ABI Prism^®^ SDS 7000 (Applied Biosystems by Life Technologies^TM^, CA, United States) to determine the location of the gene of interest *petF*, by comparing its Ct value with single-copy gene of reference. Following our previous work (Lommer et al., 2010), it was established that to accurately determine the location of the *petF* gene, two reference genes were necessary for comparison. The first selected gene was the translation elongation factor 1 alpha (*tef1a*), established as a single-copy gene by screening diverse phylogenetically unrelated genomes; this gene has a nuclear location and is used for normalization of the Ct values. The *rbcS* gene, encoding for the small subunit Ribulose-1,5-bisphosphate carboxylase oxygenase or RuBisCO, was selected as the single-copy chloroplast gene used to characterize the chloroplast location. The *18S* rRNA gene was also amplified with general primers for all *Thalassiosira* strains used in the study to confirm that all strains belonged to the *Thalassiosira* genus. The qPCR reactions (25 μl) were composed of 12.5 μl of Platinum SYBRgreen^®^ (Invitrogen by Life Technologies^TM^, Carlsbad, CA, United States), 0.5 μl of 10 μM forward and reverse primers each, 6.5 μl of PCR-H_2_O, and 5 μl of sheared gDNA template. The reactions were carried out with cycling conditions of 2 min at 50°C, 2 min at 95°C, 40 cycles of (15 s at 95°C and 30 s at 60°C), 15 s at 95°C followed by a dissociation stage of 20 s at 60°C, and a final extension of 15 s at 95°C. Triplicates of two independent biological samples per strain and no-template controls were run for each primer pair. The designed primer sets (Primer Express v2.0 by Applied Biosystems by Life Technologies^TM^, Carlsbad, CA, United States) were tested and optimized (Supplementary Table S2; Lommer et al., 2010) to detect these specific genes (amplicon of approximately 100 bp) with high amplification efficiency (>95%). The amplification efficiency of individual primer pairs was evaluated by using the LinReg program^2^ on amplification curves from assays with equal DNA amounts from diverse *Thalassiosira oceanica*, *Thalassiosira pseudonana*, and *Thalassiosira weissflogii*. Relative gene copy number was assessed using Ct, where each Ct was equal to a two-fold concentration difference. The mean Ct values of triplicate reactions of the two biological replicates at a threshold level of 0.2 were used to compare the values of the gene of interest to the ones of the genes of reference. The *petF* copy number was compared with the Ct values of either the *rbcS* or *tef1a* gene. The Ct values are presented as the mean Ct values of the triplicate values of both biological replicates for each strain studied. To obtain a clear gene location, the gene copy number was calculated by normalizing Ct value from the gene of interest to the Ct values of *tef1a* and was thereafter used to calculate the relative gene copy numbers with the formula 2^Δ*Ct*^, where the concentration fold difference is represented by ΔCt = Ct_(gene of reference)_ − Ct_(gene of interest__)_ (Pitti et al., 1998; Pelham et al., 2006). The *tef1a* gene was compared with itself, as it is known to be single-copy gene and thus yielded the standardized value of 1.

### petF, *18S*, and ITS2 PCR

The *petF*, *18S* rRNA, and internal transcribed spacer (ITS2) regions were prepared for sequencing using standard PCR composed of 2.5 μl of 10× DreamTaq buffer (Fermentas, Life Science, Thermo Fisher Scientific, Waltham, MA, United States), 1.25 μl of dNTP’s 10 mM, 2.5 μl of each 10 mM primers (Supplementary Table S2; Eurofins, Ebersberg, Germany), 0.25 μl of 5 U/μl of DreamTaq^TM^ DNA polymerase (Fermentas, Life Science, Thermo Fisher Scientific, Waltham, MA, United States). Template DNA for *petF*/*18S* rRNA gene amplification was 3 μl of DNA template and 13 μl of PCR water, while 1.5 μl of DNA template with 14.5 μl of PCR water was used for the ITS2 amplification. The *petF* PCR was carried out with cycling conditions of 30 s at 95°C, 30 cycles (30 s at 95°C, 30 s at 53°C for *T. oceanica* and at 55.8°C for *T. pseudonana*/*T. weissflogii*), 15 s at 72°C, and a final extension of 1 min at 72°C. The *18S* rRNA gene was amplified as for *petF* except with an annealing temperature of 53°C for 30 s. The ITS2 regions were treated with modified ITS cycling conditions as described in Kumar and Shukla (2005) with cycling conditions of 10 min at 95°C, 10 cycles (1 min at 95°C and 1 min at 55°C), 1.5 min at 72°C, and 10 min at 72°C. Different primers were used to assure maximum amplification efficiency of each species (Supplementary Table S2). The size of the PCR products was thereafter verified by electrophoresis on a 1.3% agarose gel to assure presence and amplification of the DNA fragment. Positive samples were sequenced.

### Sequencing

Standard 3730xl Sanger sequencing (Applied Biosystems by Life Technologies^TM^, Carlsbad, CA) of the *petF* gene, the *18S* rRNA, and the ITS1–5.*8S*–ITS2 region was carried out (Supplementary Table S2). Consensus sequences of ITS1–*5.8S*–ITS2 per strain were obtained using SEQUENCHER 5.4.1^3^, MI, United States). Representatives of the Sanger *18S* and *petF* sequences were determined with an automatic approach, as follows: the Sanger raw sequencing results for the *18S* and *petF* were mapped to *Thalassiosira* reference sequences (*18S* accessions: AAFD02000029.651044.652838, AGNL01025219.4749.6540, and FJ600728.1.1764; *petF* accessions: YP_874492.1, YP_009093409.1, and EJK54785.1) via local BLAST (Version: 2.2.28+, also tested with the more recent version 2.10.1+, “blastn -task blastn -e-value 1e-5 -max_target_seqs 1” and “blastx -e-value 1e-5 -max_target_seqs 1” for the *18S* and *petF*, respectively; Altschul et al., 1997); and the first best hit aligned region on each query sequence was extracted. If multiple sequences per species were obtained, they were further combined using the CAP3 assembly tool (Version 02/10/15; Huang and Madan, 1999); the longest resulting sequence exhibiting similarity to one of the reference sequences was picked for phylogenetic reconstructions. In case of *petF*, the reference sequences were further used to determine the reading frame for translation into amino acid sequences via the EMBOSS transeq tool (Version 6.6.0.0; Rice et al., 2000). The final sequences for phylogenetics are deposited in National Center for Biotechnology Information (NCBI) and have the following accession numbers: MN809232–MN809243 for the ITS1–*5.8S*–ITS2, MN807452–MN807463 for the *18S*, and finally, MN846055–MN846066 for the *petF*.

### Phylogenetics

The PetF protein sequences from the genus *Thalassiosira* and related species were obtained from the NCBI databases (including target peptide sequences; May 2018; Supplementary Figure S1). The downloaded sequences were used as query for similarity searches to find additional protein homologs of species annotated as Thalassiosirales in the marine microbial eukaryote transcriptome project (MMETSP; Keeling et al., 2014). Best BLAST hits (e-value cutoff 10^–10^) were extracted to determine candidate homologs and to define a representative protein sequence set. In some cases, amino acids were removed at the end or the beginning as likely incorrectly predicted N- or C-termini as revealed by multiple sequence alignments (see below). Here, we noticed that some protein sequences were not starting with methionine residues, while covering more than the full length of the NCBI *Thalassiosira* reference sequences (see above). We trimmed them from the start until the occurrence of the first “M” residue. Further, one “X” residue was trimmed from the C-terminus to adjust it to the other sequence ends for the same genus (see Supplementary Figure S1 for details on trimmed residues). Two distinct *petF* homologs found for *Minutocellus polymorphus* were discarded because we were not able to differentiate clearly if they represented two diversified gene copies or contamination by another diatom species (accessions: CAMPEP_0197733592 and CAMPEP_0197725580). All sequence alignments were reconstructed using MAFFT tool v7.123b (options: “–maxiterate 1000 –localpair”; Katoh and Standley, 2013). First, the NCBI homologs were aligned, followed by the gradual addition of homologs derived from MMETSP and from the Sanger sequencing via the “–add” and “–addFragments” alignment options, respectively. Finally, phylogenetic trees were produced from alignments using IQ-TREE 1.5.5 with 1,000 non-parametric bootstrap replicates and the ModelFinder function enabled (Inferred model of substitution: WAG+G4; Nguyen et al., 2015). An alternative phylogeny without target peptides was reconstructed after removal of the first 48 alignment columns that were found to cover potential target peptide sequences. The *18S* phylogeny of the species sequence set corresponding to the PetF proteins was obtained from the SILVA sequences database (May 2018; Quast et al., 2013) and from sequences provided by the MMETSP project for individual eukaryotic transcriptomes. The *18S* of *Conticribra weissflogiopsis* (accession: KT347147.1.3358) was additionally included because it was found to be the most closely related to the *18S* of *T. oceanica* CCMP1616 as determined by the SILVA webservice “Search and classify” tool (min. identity: 0.95; number of neighbors: 1). Phylogenies for the *18S* sequences were reconstructed with MAFFT and IQ-TREE applying the same parameters as for the PetF proteins (inferred model of substitution: TIM3+F+R2). First, the *18S* sequences from databases were aligned with the “–maxiterate 1000 –localpair –adjustdirection” options of MAFFT, and Sanger sequencing results were added (MAFFT; options: “–addFragments”). The ITS1–*5.8S*–ITS2 phylogeny was produced directly from the consensus sequences (inferred model of substitution: HKY+F+G4) with *Phaeodactylum tricornutum* as the outgroup (accession: EF553458.1). Alignments and phylogenetic trees were uploaded to FigShare (figshare.com; doi: 10.6084/m9.figshare.12783209). To determine if the *petF* gene is encoded on plastid genomes of other algae, the GenBank annotations of all 2,670 RefSeq plastid genomes (May 2018; O’Leary et al., 2016) were downloaded and searched for *petF* genes (string search: “gene = *PetF*”). Algal taxa known to harbor plastids of green algal origin (i.e., ‘Viridiplantae,’ ‘Euglenozoa,’ ‘Rhizaria,’ and ‘*Lepidodinium*’) were excluded (Supplementary Table S1).

## Results

### Transfer of *petF* Gene Was Uniquely Found in the *Thalassiosira oceanica* Strains

**FIGURE 1:**
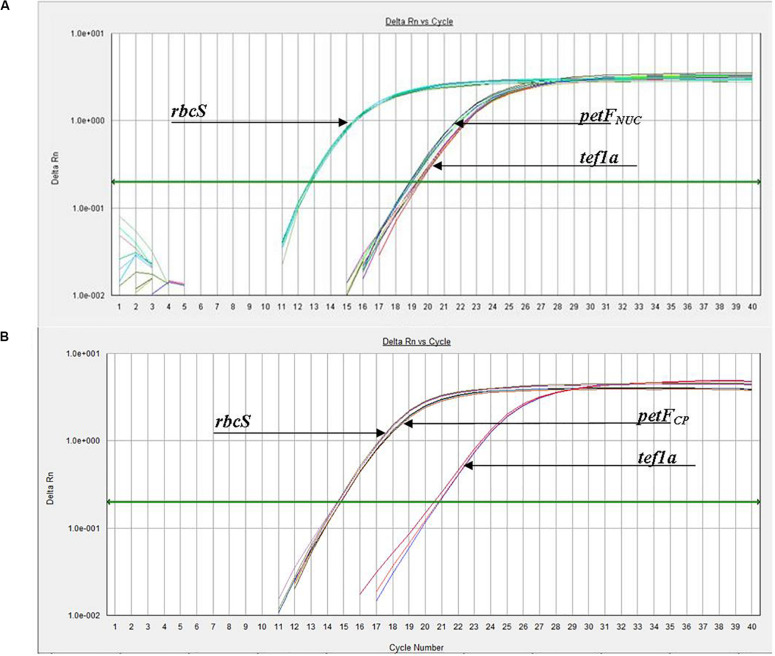
Experimental amplification curves (qPCR) for *petF*, *tef1a*, and *rbcS* genes using whole genomic DNA from **(A)** *Thalassiosira oceanica* (e.g., CCMP1006) and **(B)** *Thalassiosira weissflogii* (e.g., CCMP1049), demonstrating examples of *petF* transfer to the nuclear genome and chloroplast genome retention, respectively. **(B)** Arrowhead of *rbcS* is pointing at the group of lines slightly to the left, while the *petF* arrowhead is pointing to the group of lines to the right.

**FIGURE 2:**
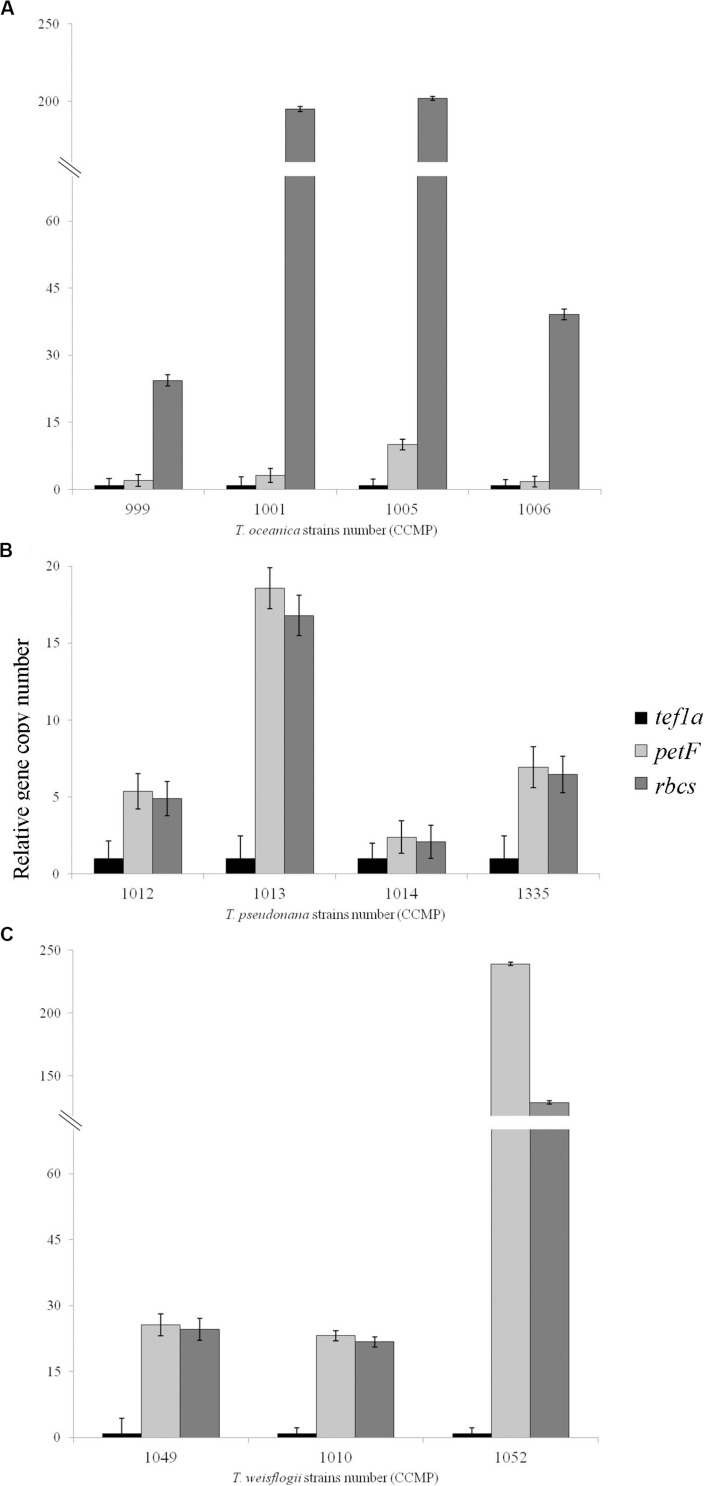
Relative gene copy number of the gene of interest *petF* in comparison with the genes of reference with either a chloroplastic (*rbcS*) or nuclear location (*tef1a*). These values were obtained using 2^Δ*Ct*^ ± SE for **(A)** *Thalassiosira oceanica*, **(B)** *Thalassiosira pseudonana*, and **(C)** *Thalassiosira weissflogii*. The standard error on the mean Ct values is overlapping in all the cases where the differences were not significant and is not overlapping when these values are significantly different (Table 2), as expected for such significance and thus confirms the gene location highlighted via qPCR. Amplification for *Thalassiosira oceanica* CCMP1616 failed with all primers.

We applied a qPCR assay on multiple strains of three *Thalassiosira* spp., namely, *Thalassiosira oceanica*, *Thalassiosira pseudonana*, and *Thalassiosira weissflogii*. Examples of the results obtained for *petF*_NUC_ and *petF*_CP_ localization are presented in Figure 1 where the *petF* location is determined by comparing its gene copy number with the gene copy numbers of the nuclear and chloroplast reference genes from the same DNA extract. Details of the location of the *petF* gene for each strain and associated statistical tests are presented in Table 2. None of the no-template controls run for each strains had a signal. Within *T. oceanica*, five strains (CCMP0999, 1001, 1005, 1006, and 1616) were tested; and in all strains except CCMP1616, the *petF* gene copy number corresponded more closely to the copy number of the nuclear gene *tef1a* rather than to the copy number of the chloroplast-encoded *rbcS* gene, corroborating transfer to the nuclear genome (Figure 2 and Table 2). In CCMP0999 and 1005, the estimated copy numbers for the *petF* gene were statistically significantly different than both *rbcS* and *petF* gene copy numbers. However, the difference was much greater with *rbcS*, thus supporting a transfer of the *petF* gene to the nuclear genome, as demonstrated in Lommer et al. (2010). The qPCR results were not conclusive for *T. oceanica* CCMP1616 because the gene could not be amplified by any of the primers used for all of the other *Thalassiosira* strains from this study. The CCMP1616 strain originated from the Mediterranean Sea, whereas the four other strains were isolated from the open waters of the North Atlantic. Based on the qPCR assays, the strains of *T. pseudonana* (CCMP1012, 1013, 1014, and 1335) and *T. weissflogii* (CCMP1010 and 1049) were all confirmed to have retained the *petF* gene in the chloroplast genome (Figure 2 and Table 2). The qPCR assays conducted for *T*. *weissflogii* CCMP1052 resulted in *petF* gene copy numbers that were significantly different to both reference genes; however, the *petF* and *rbcS* (chloroplast) relative copy numbers are much higher than the gene copy number of *tef1a* (nuclear), suggesting that the *petF* is located in the chloroplast in keeping with the location for this species.

**TABLE 2:** *petF* location status (Status) based on relative copy numbers (Copy #) ±SE and paired two-sample *t*-test for means Ct values ($\bar{x}$ Ct) of amplification curves ±SD, where mean Ct values are from triplicate qPCRs of two biological replicates.

Species	Strain	Gene	$\bar{x}$ Ct	SD	*p* value	Copy #	SE	Status
*Thalassiosira*	0999	*tef1a*	19.2	0.5	**4.64E–03**	1	1	
*oceanica*		*petF*	18.2	0.1	-	2	1	Nuclear transfer
		*rbcS*	14.6	0.1	**2.12E–09**	24	1	
	1001	*tef1a*	23.1	0.9	1.48E–02	1	2	
		*petF*	21.5	0.3	-	3	2	Nuclear transfer
		*rbcS*	15.5	0.4	**8.81E–10**	195	2	
	1005	*tef1a*	19.9	0.4	**2.83E–06**	1	1	
		*petF*	16.6	0.1	-	10	1	Nuclear transfer
		*rbcS*	12.2	0.2	**9.49E–09**	202	1	
	1006	*tef1a*	19.6	0.3	1.04E–02	1	1	
		*petF*	18.8	0.1	-	2	1	Nuclear transfer
		*rbcS*	14.3	0.0	**3.20E–08**	39	1	
	1616	*tef1a*	No amplification					
		*petF*	No amplification					Unknown
		*rbcS*	Amplification with T.p/T.w primers					
*Thalassiosira*	1012	*tef1a*	17.9	0.2	**1.21E–08**	1	1	
*pseudonana*		*petF*	15.5	0.2	-	5	1	Plastid retention
		*rbcS*	15.6	0.1	1.95E–01	5	1	
	1013	*tef1a*	18.6	0.5	**2.67E–05**	1	1	
		*petF*	14.4	0.2	-	19	1	Plastid retention
		*rbcS*	14.5	0.1	1.80E–01	17	1	
	1014	*tef1a*	16.7	0.2	**5.50E–07**	1	1	
		*petF*	15.1	0.6	-	2	1	Plastid retention
		*rbcS*	15.4	0.5	8.48E–02	2	1	
	1335	*tef1a*	16.8	0.8	**1.97E–04**	1	1	
		*petF*	14.0	0.6	-	7	1	Plastid retention
		*rbcS*	14.2	0.3	6.58E–01	6	1	
*Thalassiosira*	1010	*tef1a*	18.8	0.2	**1.15E–07**	1	1	
*weissflogii*		*petF*	14.2	0.0	-	23	1	Plastid retention
		*rbcS*	14.3	0.1	1.79E–02	22	1	
	1049	*tef1a*	19.6	1.2	**2.70E–05**	1	3	
		*petF*	14.9	0.5	-	26	3	Plastid retention
		*rbcS*	14.9	0.5	2.40E–01	25	3	
	1052	*tef1a*	19.2	0.2	**1.23E–13**	1	1	
		*petF*	11.2	0.2	-	239	1	Plastid retention
		*rbcS*	12.1	0.3	**9.09E–07**	129	1	

*Differences between petF and tef1a/rbcS (references) are significant when p < 0.01 (bold).*

### Phylogenetic Affiliation of the *Thalassiosira* Strains

The *petF* gene transfers determined via the qPCR assays were further analyzed in the context of the phylogenetic relationships within the Thalassiosirales. Maximum likelihood trees of the ITS2 region, the PetF proteins, and the *18S* rRNA gene were constructed for each experimentally studied strain and their closely related species. The highly intraspecific discriminating sequences of the ITS2 region were sequenced to provide a more refined classification of the strains within the *Thalassiosira* genus and to confirm their detailed taxonomic relationship (Amato et al., 2007; Moniz and Kaczmarska, 2009; Sorhannus et al., 2010). Classification on the basis of the ITS2 indicated a clear separation of the three *Thalassiosira* species studied where *T. oceanica* and *T. weissflogii* formed a monophyletic clade (Supplementary Figure S2). However, the ITS2 sequences from strains of the same species were nearly identical and therefore could not be distinguished on the sole basis of the diversity of their ITS2 region. *T. oceanica* CCMP1616 was the only outlier, which appeared to branch deeply in comparison with the other *T. oceanica* strains.

**FIGURE 3:**
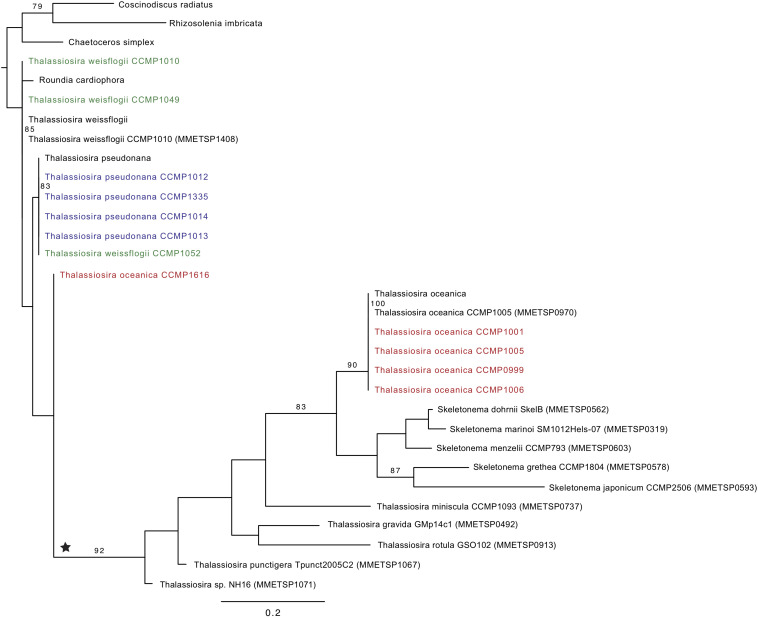
Phylogenetic tree of ferredoxin (PetF) protein sequences of experimentally cultured *Thalassiosira* (colored) where *Thalassiosira weissflogii* CCMP1052 clusters within the *Thalassiosira pseudonana* cluster and *Thalassiosira oceanica* CCMP1616 outside the *T. oceanica* cluster, resulting in unresolved positions of these strains. Bootstrap values above 70 are presented in this tree, and the outgroup root clade contains *Rhizosolenia imbricata*, *Coscinodiscus radiatus*, and *Chaetoceros simplex*. The ★ symbol marks a putative *petF* gene transfer event. For details on the sequences added from public databases, see Supplementary Figure S1.

**FIGURE 4:**
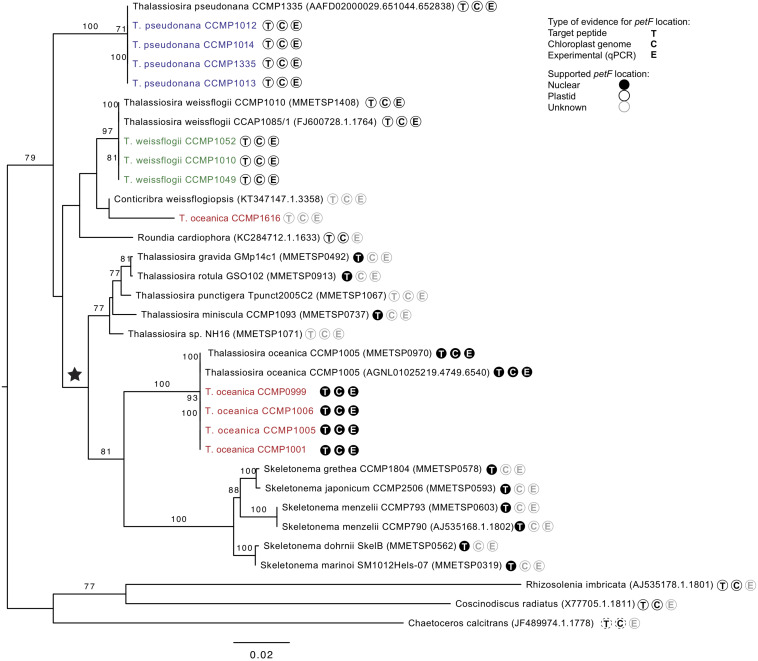
Interspecific phylogenetic tree of genus *Thalassiosira* based on *18S* gene sequences from databases and all experimentally tested strains sequenced (colored) with one clade containing *Rhizosolenia imbricata*, *Coscinodiscus radiates*, and *Chaetoceros calcitrans* as outgroup root. Parametric bootstraps values above 70 are presented at the branches. The ★ symbol marks the putative event of *petF* gene transfer to the nuclear genome. Further symbols at the tree leaves indicate the availability of evidences on the location of the *petF* gene considering at least one representative of the same species. T: Target peptide present or absent indicating nuclear or plastid gene location, respectively. C: Gene present or absent from the chloroplast genome annotations indicating plastid or nuclear gene location, respectively. E: Experimentally verified plastid location based on qPCR. Evidences available for *Thalassiosira oceanica* were not applied to *T. oceanica* CCMP1616, as it does not form a monophyletic clade with the other strains of the same species. Dashed lines shown for *C. calcitrans* denote evidences based on a different species of the same genus (=*Coscinodiscus radiatus*).

The phylogenetic reconstruction of PetF amino acid sequences showed that, with the exception of *T*. *pseudonana* CCMP1616 and *T*. *weissflogii* CCMP1052, the experimentally cultured strains of *T. oceanica*, *T. pseudonana*, and *T. weissflogii* grouped with *petF* genes from annotated genome sequences of the corresponding species (Figure 3). Here, we extended our analysis by including additional *petF* homologs from the genera *Thalassiosira* and *Skeletonema* obtained via transcriptome assemblies from the marine microbial transcriptome project (Marine Microbial Eukaryotes Transcriptome Project; MMETSP; Keeling et al., 2014). In the resulting phylogenetic tree (Figure 3), *T. pseudonana* and *T. weissflogii* CCMP1052 formed a close monophyletic relationship, albeit without bootstrap value support. Two strains of *T. weissflogii* were placed next to two database homologs for this species and one other member of Thalassiosirales (*Roundia cardiophora*). However, branch lengths here were zero, and there was no monophyletic clade that united all of the four taxonomic units of *T. weissflogii*. One strain of *T. weissflogii* (CCMP1052) grouped with *T. pseudonana*, while the *T. pseudonana* clade itself is a sister group to remaining species of the genus *Thalassiosira*. The latter comprised a highly supported monophyletic subclade (bootstrap support 92%) containing multiple *Skeletonema* spp., *Thalassiosira* spp., and four of the experimentally cultivated *T. oceanica* strains (CCMP0999, 1001, 1005, and 1006). As expected from the ITS2 phylogenetic tree results (Supplementary Figure S2), the *PetF* amino acid sequence of *T. oceanica* CCMP1616 formed a separate branch (Figure 3) from the other *T. oceanica* strains. The elevated evolutionary rates (i.e., branch lengths) estimated from the tree branch lengths connecting the root with *T. oceanica* CCMP1616 (0.09 substitutions per site) compared with the branch distance of *T. oceanica* CCMP1616 to its sister clade comprising the other isolates of *T. oceanica* and the genus *Skeletonema* (0.18 substitutions per site) indicate for an evolutionary transition. As previously observed (Groussman et al., 2015), most of the recovered *petF* transcript homologs of the mixed *Thalassiosira–Skeletonema* clade coded for a target peptide at the N-terminus (Supplementary Figure S1), indicating plastid-targeted proteins encoded in the nuclear genome and synthesized in the cytoplasm (Gould et al., 2008). Target peptides were not identified for the *PetF* amino acid sequences of *Thalassiosira punctigera* or *Thalassiosira* sp. strain NH16, but their N-termini appeared incomplete as they were derived from transcriptome rather than genome sequences and lacked the methionine start codon. The observed differences in evolutionary rates cannot be explained by the presence or absence of target peptides as demonstrated by another reconstructed PetF phylogeny after removal of alignment columns covering potential target peptide sequences (Supplementary Figure S3). The complete PetF amino acid sequence is short (roughly 100 amino acids long) and on its own not suitable to uncover detailed phylogenetic relationships. To validate the results regarding *petF* and to obtain a large-scale resolution of the *Thalassiosira* taxon, an *18S* rRNA phylogenetic tree was reconstructed based on sequences obtained from the species and strains used in this study (Figure 4), together with additional species from public databases. The resulting phylogeny showed three distinct and highly supported clades formed by each of the species of interest in our study plus additional species of the order Thalassiosirales. Although the overall *18S* rRNA phylogeny resembled that obtained for *PetF*, the differences in varying branch lengths of the clades were not observed as in the PetF tree. Nucleotide substitutions in rRNA genes and amino acid substitutions in proteins are not expected to behave the same. However, the overall relative branch lengths considering the same clades should be comparable. This suggests that the differences in evolutionary rates observed for trees of the *18S* rRNA genes and the PetF proteins were not simply caused by overall difference in genome evolution for the different species compared. Another notable difference between the *18S* and *PetF* topologies was the monophyletic grouping of the additional *Thalassiosira* species, other than *T*. *pseudonana*, *T*. *weissflogii*, or *T*. *oceanica*. The only sequence not clustering according to its currently assigned taxonomy was *T. oceanica* CCMP1616. This strain formed a clade with another species (*Conticribra weissflogiopsis*) within the Thalassiosirales. Although this grouping was not supported by the bootstrap analysis, these results suggest that *T. oceanica* CCMP1616 is more related to other species within the order Thalassiosirales than to other strains classified as *T. oceanica*.

### *petF* Genes of Algal Plastid Genomes

All available genome annotations of plastids sequences for *petF* (RefSeq, May 2018; Pruitt et al., 2012) were searched to find additional *petF* gene transfer events in the evolution of diatoms and other algae (Supplementary Table S1). Here, we interpreted the absence of the *petF* gene in the plastid genome as supporting evidence for functional transfer to the nuclear genome. For the 21 diatoms (Bacillariophyta) plastid genomes available for screening, only *T*. *oceanica* CCMP1005 lacked a plastid-encoded *petF* gene (Lommer et al., 2010). Furthermore, we considered the complete 43 species set available for the Stramenopiles taxon with plastid genomes of diatoms and other photosynthetic eukaryotes. In addition to *T*. *oceanica* CCMP1005, we found that *petF* was missing in the plastid genomes of two members of Pelagomonadales (*Aureococcus anophagefferens* and *Aureoumbra lagunensis*) as well as in *Pleurocladia lacustris*. Based on our assumptions on the distinct taxonomic classes assigned to those species (Supplementary Table S1), only the two Pelagophyceae here would form a monophyletic group. All other species share a last common ancestor (LCA) with species from the same class that have a plastid-encoded *petF* gene. Thus, as we do not expect the *petF* gene to be transferred from the nucleus to the plastid genome, the most parsimonious explanation for the current observation would lead to at least three independent *petF* gene transfers (i.e., once in each of the classes Pelagophyceae, Coscinodiscophyceae, and Phaeophyceae). In order to obtain a broader sense on the *petF* gene location, we searched 146 plastid genomes representing other eukaryotic groups beyond the Stramenopiles taxon; only green plants were excluded as the *petF* gene is generally known to have been transferred to the nuclear genome (the Plantae Chlorophyta group; Martin et al., 1998, 2002). A *petF* annotation was not found in any of the plastid genomes of the searched (Supplementary Table S1) Haptophyceae, non-photosynthetic Apicomplexa, two closely related photosynthetic species of Chromerida (*Chromera velia* and *Chromerida* sp. RM11), and one species of Rhodophyta (*Platysiphonia delicata*).

## Discussion

### Phylogenetic Classification

Our phylogenetic analyses confirm that all of the Thalassiosirales species studied here clustered together in one monophyletic group, supporting one common ancestor within the diatoms (Sabir et al., 2014). Our phylogenetic representations (Figures 3, 4) showed that the *Skeletonema* genus is nested within *Thalassiosira* as supported by previous studies (Medlin et al., 1993; Alverson et al., 2007; Chappell et al., 2013; McQuaid et al., 2018), indicating that the genus *Thalassiosira* is paraphyletic. The branch length between species in the phylogenetic trees presented (Supplementary Figure S2, Figures 3, 4) can be considered to represent genetic differences. While the tree topologies provide contradictory evidences on the exact relationship of the three species (*T. pseudonana*, *T. weissflogii*, and *T. oceanica*), their level of genetic divergence (Figures 3, 4) is congruent with previous demonstration that *T. pseudonana* and *T. weissflogii* are more closely related to each other than to *T. oceanica* (Luddington et al., 2012). This observation coupled with our knowledge of the distinct environmental conditions in which these species are detected (Table 1) reinforces the hypothesis that environmental factors influenced diatom speciation (Whittaker et al., 2012) and were possibly key for speciation in the *Thalassiosira* genus. The relative branching in the rooted phylogenies (Figures 3, 4) indicates that most strains of *T. oceanica* share a more recent LCA with the genus *Skeletonema* (and some additional *Thalassiosira* species) than the LCA shared with either *T. pseudonana* or *T. weissflogii*. Thus, this indicates that the LCA of *T*. *oceanica* and the *Skeletonema* lineage diversified after the LCA shared with *T*. *pseudonana* or *T*. *weissflogii*. It should be noted that the available genetic information for most species of the genus *Thalassiosira* is still scarce, and therefore our analyses were limited to the available species, leaving a degree of uncertainty regarding the complete phylogenetic relationship within the genus *Thalassiosira*.

We question the taxonomic identity of the strain classified as *T. oceanica* CCMP1616, after the *petF* gene failed to amplify with the primers used for the other *T. oceanica* strains (Figure 2). The classification of this strain with the well-established eukaryotic barcoding region ITS2 (Supplementary Figure S2; Coleman, 2003, 2009; Sorhannus et al., 2010; Caisova et al., 2011; Senapin et al., 2011; Whittaker et al., 2012) showed that CCMP1616 is unlikely to belong to the *T. oceanica* species because it is not part of the highly supported monophyletic *T. oceanica* clade. The *18S* phylogeny further indicates that it instead stands as an independent neighbor of the other *Thalassiosira* clades, which cluster well according to their assigned species (Figure 4). Instead, our results suggest that the CCMP1616 strain may more likely be affiliated to *Conticribra weissflogiopsis*. We further compared our results with the *5.8S* and ITS phylogenetic trees drawn by von Dassow et al. (2008), confirming that most tested strains formed clusters within their respective monophyletic groups as previously established, except for *T. oceanica* CCMP1616. Another study also used ITS2 to differentiate shifts in *Thalassiosira* spp. compositions and identified *T. oceanica* CCMP1616 as a different species, although it is morphologically similar to *T. oceanica* (Chappell et al., 2013). The results thus support that *T. oceanica* CCMP1616 is not part of the *T. oceanica* monophyletic group and was most likely misclassified (von Dassow et al., 2008). However, only further genomic analysis will reveal whether strain CCMP1616 belongs to *T*. *oceanica* or another species.

### Gene Transfer

The *petF* gene is typically found in the chloroplast of the Rhodophyta, Glaucophyta, Cryptophyta, and Bacillariophyceae (diatoms), whereas some other groups, Alveolata, Chlorophyta, and Streptophyta, have this gene in their nuclear genome (Martin et al., 2002; Lommer et al., 2010). The groups with nuclear *petF* have smaller chloroplast genomes ranging from ∼30 to 100 protein genes versus ≈ ≥ 140 for the ones that retained *petF* in the chloroplast (Kleffmann et al., 2004; Lommer et al., 2010). This gene content reduction is believed to result from gene loss during gene transfer causing genomic rearrangements or just by the transfer of the genes themselves (Lommer et al., 2010; Leister, 2005). Indeed, when insertion of organellar DNA to the nucleus happens, major restructuring is expected to follow (Timmis et al., 2004; Leister, 2005). Stramenopiles (including Bacillariophyceae) typically have a *petF*_CP_. However, it was previously discovered that in *T. oceanica* CCMP1005, a diatom with the typically large chloroplast genomic content of Stramenopiles (≈ 140 chloroplastic genes), the *petF* gene was relocated in the nuclear genome and replaced by *orf127* in the chloroplast genome (Lommer et al., 2010). It was suspected that the gene transfer may have been facilitated by the intrinsic features of the *petF*_CP_ gene flanking regions. Large-scale chloroplast genome reduction should also benefit from an improved regulation of the transferred genes (Woodson and Chory, 2008) because it is known that the transfer process could possibly keep the genes in both places, creating a duplicate gene. The latter would raise a more complicated management of gene expression regulation and the protein level control as well (Oudot-Le Secq et al., 2007; Woodson and Chory, 2008). It is speculated that the relocation of *petF* could be connected to the intermittent regulation and use of *fld1A* and *petF* (LaRoche et al., 1995; McKay et al., 1999; Lommer et al., 2010; Whitney et al., 2011). Indeed, when Fe is limited, *fld1A* is up-regulated in many species of diatoms to support a continued efficient control of electron transfer for photosynthesis (LaRoche et al., 1995). It was demonstrated that gene relocation from the chloroplast to the nucleus increases with environmental stress, particularly with heat in plants (Wang et al., 2012). The association between higher heat and gene transfer is further supported by the suggestion that open chromatin regions, sites where such transfers may happen, have increased accessibility when cells are subjected to temperature stress (Wang and Timmis, 2013). A similar scenario can be envisioned for unicellular algae, especially for diatoms that inhabit marine oligotrophic surface waters subject to stress caused by high light intensities and nutrient limitation. However, this type of plastid-to-nucleus transfer is only possible in organisms containing multiple plastids (Diner et al., 2017). Furthermore, the genomic DNA released by chloroplast lysis is considered a very important source for nuclear genome renewal or adaptation (Lommer et al., 2010; Diner et al., 2017). The genomic analysis of the *petF* gene transfer in different species is valuable by shedding light on the possible evolutionary processes that such chloroplast-to-nucleus transfers and their regulation imply. Indeed, Woodson and Chory (2008) argued that plastid-to-nucleus gene transfers are advantageous because, when achieved as demonstrated for *T. oceanica*, they allow simpler genome regulation. The possible logistical challenges of controlling separated genome systems (organellar versus nuclear) by the cell are therefore avoided. The results from the qPCR assays and phylogenetic trees, together with the presence or absence of target peptide in the ferredoxin amino acid sequences, showed that the *petF* gene of all *T. oceanica* strains tested (except CCMP1616) was located in the nuclear genome, whereas the *petF* gene of all tested *T. pseudonana* and *T. weissflogii* strains was located in the chloroplast genome. The location of the *petF* gene in three strains (*T. oceanica* CCMP1005, *T. pseudonana* CCMP1014, and *T. weissflogii* CCMP1052) based on qPCR results was further clarified by the *PetF* phylogenetic tree that showed that these three strains clustered within their respective group (Figure 3). Further confirming the transfer to the nuclear genomes, the *petF* genes of several strains within this monophyletic clade (*T. oceanica* and *Skeletonema* sp.; Supplementary Figure S1) contained DNA sequences coding for a target peptide needed to target the protein to the chloroplast after it is synthesized in the cytoplasm. Although the full gene sequence for the *petF* was not available for all of the species we studied, the presence of target peptides in the pre-PetF protein is an additional confirmation of transfer from the plastid to the nuclear genome. Furthermore, mutation rates of nuclear genomes are expected to be at least twice as high as the mutations rates of plastid genomes (Wolfe et al., 1987; Smith, 2015), which is congruent with a recent study on the diatom *Phaeodactylum tricornutum* (Krasovec et al., 2019).

The increase in the number of amino acid replacements represented by the length of the branches at the beginning of the *T. oceanica* CCMP1616 branch (with respect to the clade including *Thalassiosira punctigera* and *Thalassiosira* sp. strain NH16) suggests that the *petF* gene transfer happened there (Figure 3). This increased evolutionary rate is congruent with the *petF* gene relocation to the nucleus. Therefore, the *18S* rRNA gene and *petF* phylogenetic trees support a scenario where the *petF* gene transfer took place before diversification of the members of this clade from the species of *T. oceanica* and the genus *Skeletonema* (Figures 3, 4). In conclusion, we were able to trace the *petF* gene transfer back in the ancestry of the genus *Thalassiosira* after speciation from the common ancestors with *T. pseudonana* and *T. weissflogii*. The gene arrangement of these two species was reported to represent the ancestry of the Thalassiosirales order (Sabir et al., 2014) and reinforced our findings on the functional transfer of *petF*. The change in the evolutionary rate further suggests that CCMP1616 diverged prior to the transfer event (Figure 3). Given the basal positioning in relation to the transferred homologs, one could speculate that CCMP1616 may represent an intermediate state, where the *petF* was transferred to the nucleus but not yet affected by the evolutionary rate of the nucleus. The inclusion of the *Skeletonema* genus and other species of the genus *Thalassiosira* in our results strengthens the previous observations on *petF* being transferred in the *Thalassiosira* genus and being more ancient than previously reported (Lommer et al., 2010; Lommer et al., 2012). The *petF* gene was first found to have a nuclear homolog in the Chlorophyta-Plantae group after differentiation from their cyanobacterial ancestor (Martin et al., 1998). We confirm additional independent loss events amongst diatoms and other organism groups. Episodic evolution via multiple loss or independent acquisition is common in diatom adaptation (Theriot et al., 2015; Diner et al., 2017), as also seen with the *psb28* gene (Jiroutova et al., 2010). The search in plastid genome annotations (Supplementary Table S1) highlighted the absence of the *petF* genes from several plastid genomes (Haptophyceae, non-photosynthetic Apicomplexa, *Chromera velia*, *Chromerida* sp. RM11, and *Platysiphonia delicata*). This absence suggests that the *petF* gene transfer from the chloroplast to the nucleus may have happened several times in evolutionary history. This aspect reinforces the hypothesis that the *petF* gene transfer occurred following environmental pressure and adaptation to different biogeochemical conditions, e.g., Fe limitation for phytoplankton (Chisholm et al., 2001; Moore et al., 2004; Boyd et al., 2007). In conclusion, these results indicate that although the transfer of *petF* to the nucleus is not essential to sustain a photosynthetic lifestyle, repeated endosymbiotic gene transfer of the *petF* supports an evolutionary benefit for photosynthetic eukaryotes.

Organellar DNA transfers were demonstrated to be continuously in progress and more frequent than anticipated (Fujita et al., 2008; Kleine et al., 2009) even if only a handful of research proved such transfer (chloroplast to nucleus in transgenic tobacco plant and mitochondrion to nucleus in yeast (Thorsness and Fox, 1990; Huang et al., 2003; Stegemann et al., 2003). Further evidences showed that high temperature could be the driving condition for functional gene transfer (Wang et al., 2012). Indeed, temperature stress was connected to the increased accessibility of chromatin regions, which were identified as the regions where successful organelle-to-nucleus transfers take place (Wang and Timmis, 2013). In the present *Thalassiosira* spp. case, the investigated species thrive in two different environmental temperatures. *Thalassiosira oceanica* grows in warmer water of 22–26°C, as does some *Skeletonema* species (e.g., Skeletonema costatum and Skeletonema ardens Yan et al., 2002; Kaeriyama et al., 2011), whereas *T. pseudonana* and *T. weissflogii* grow in 11–16°C water. This difference, but mostly the increase of temperature initially experienced by *T. oceanica* and possibly by some *Skeletonema* species, might have created the type of stress related to the improved accessibility of chromatin. Temperature and the exposition to different Fe concentration could have led to the functional *petF* transfer in *T. oceanica* and may have contributed to *T. oceanica* being remarkably tolerant to low Fe concentration compared with the other two Thalassiosira species studied here (Lommer et al., 2012). The reasons for successful organellar gene transfer are still unknown and need to be further investigated; however, some connections to ecological advantages through evolution are made. This capacity to adapt or even just to acclimate to a new environment will directly influence the composition of marine communities and their effect on the oceanic ecosystems (OceanStudiesBoard, 2010). Diatoms are well known for their ability to adapt successfully to new environmental conditions (von Dassow et al., 2008); and since one of the most successful diatom classes is *Thalassiosira* (Round, 1990; Kaczmarska et al., 2006), their response to environmental triggers to form great blooms in any oceanic water around the globe must be understood to predict their response to major environmental changes (e.g., ocean acidification and CO_2_ sequestration).

## Conclusion

The present study shows that although the taxonomic assignment of cultured strains within three species of the *Thalassiosira* genus was confirmed, the taxonomy of the strain CCMP1616 could not be confidently assigned to *Thalassiosira oceanica*. Within these three closely related species (*T. oceanica*, *Thalassiosira pseudonana*, and *Thalassiosira weissflogii*), only *T. oceanica* diversified after the functional *petF* gene transfer from chloroplast to nuclear genome. It also demonstrates that the simple qPCR assay designed for this study is an appropriate tool for determining such gene transfer in closely related species and strains for which complete genome information is not available, thus providing insight into the evolutionary processes of chloroplast genome reduction. Our results showed that the *petF* transfer did not occur in *T. pseudonana* and *T. weissflogii* and confirms that the *petF* gene transfer happened after the differentiation of these three species. Moreover, additional species of the genus *Thalassiosira* and the genus *Skeletonema* expand the species exhibiting nuclear-encoded *petF* genes. The *petF* gene transfer occurred only once in the evolution of diatoms, but multiple times considering other closely related photosynthetic lineages. We speculate that the high tolerance of *T. oceanica* to severe Fe limitation and its ability to grow at higher temperature than other Thalassiosirales may be drivers for such transfer and that elevated temperatures might have increased the accessibility of chromatin, promoting the exchange of DNA as suggested by Wang and Timmis (2013). This hypothesis further supports that the functional transfer of the *petF* gene to the nuclear genome in *T. oceanica* may have originated from *T. oceanica*’s preference for higher temperature, coupled with chronically low Fe concentrations in warm, oligotrophic surface waters.

## Data Availability Statement

The datasets generated for this study can be found in the NCBI, MN809232-MN809243 for ITS1-5.8S-ITS2, MN807452-MN807463 for 18S, and MN846055-MN846066 for PetF. Alignments and phylogenetic trees were uploaded to FigShare (figshare.com; doi: 10.6084/m9.figshare.12783209).

## Author Contributions

A-SR and JLR designed the experiments conducted by A-SR. CW designed and performed the phylogenetics analyses. All authors analyzed and interpreted the data. A-SR and CW wrote the manuscript with contribution by JLR.

## Conflict of Interest

The authors declare that the research was conducted in the absence of any commercial or financial relationships that could be construed as a potential conflict of interest.

## Footnotes

1 http://ncma.bigelow.org

2 http://www.gene-quantification.de/

3 https://www.genecodes.com/

## Supplementary Material

Supplementary Figure S1 Peptide homologs showing target peptide at the N-terminus typically found for plastid-targeted proteins encoded on the nucleus. A label indicates if the target peptide is suspected to be present; for sequences 8 & 9 information about the targeting peptide is missing. If protein sequences from public databases were modified for the alignment, the legend exhibits information on the details (See ‘Note:’).

Supplementary Figure S2 ITS2 phylogenetic tree of experimentally cultured *Thalassiosira* (colored) with *Phaeodactylum tricornutum* as outgroup root; where only bootstraps values above 70 are presented.

Supplementary Figure S3 Phylogenetic tree of ferredoxin (PetF) protein sequences without target peptides where experimentally cultured *Thalassiosira* strains are colored. Parametric bootstraps values above 70 are presented at the branches. The outgroup root clade contains *Rhizosolenia imbricata*, *Coscinodiscus radiatus*, and *Chaetoceros simplex*. For details on the sequences added from public databases see Supplementary Figure S1.

Supplementary Table S1 Representation of the petF gene in plastid genomes of algae not considering green plants.

Supplementary Table S2 Oligonucleotides primers used for qPCR analysis 1 (gray shading) and for Sanger sequencing.

## Abbreviations

Ct: threshold cycle
Fe: iron
ITS: internal transcribed spacer
LCA: last common ancestor
*petF*: ferredoxin
*petF*_CP_: chloroplastic genome *petF*
*petF*_NUC_: nuclear genome *petF*
qPCR: quantitative polymerase chain reaction
*rbcS*: small subunit of Ribulose-1,5-bisphosphate carboxylase oxygenase or RuBisCO
*tef1a*: translation elongation factor 1 alpha
*iron*: Fe.

## References

[B1] Altschul S. F. Madden T. L. Schäffer A. A. Zhang J. Zhang Z. Miller W. (1997). Gapped BLAST and PSI-BLAST: a new generation of protein database search programs. *Nucleic Acids Res.* 25 3389–3402. 9254694 10.1093/nar/25.17.3389 PMC146917

[B2] Alverson A. J. Jansen R. K. Theriot E. C. (2007). Bridging the Rubicon: phylogenetic analysis reveals repeated colonizations of marine and fresh waters by thalassiosiroid diatoms. *Mol. Phylogenet. Evol.* 45 193–210. 10.1016/j.ympev.2007.03.024 17553708

[B3] Amato A. Kooistra W. H. C. F. Levialdi Ghiron J. H. Mann D. G. Pröschold T. Montresor M. (2007). Reproductive isolation among sympatric cryptic species in marine diatoms. *Protist* 158 193–207. 10.1016/J.PROTIS.2006.10.001 17145201

[B4] Archibald J. M. (2009). The puzzle of plastid evolution. *Curr. Biol.* 19 R81–R88. 10.1016/J.CUB.2008.11.067 19174147

[B5] Armbrust E. V. (2009). The life of diatoms in the world’s oceans. *Nature* 459 185–192. 10.1038/nature08057 19444204

[B6] Bock R. Timmis J. N. (2008). Reconstructing evolution: gene transfer from plastids to the nucleus. *Bioessays* 30 556–566. 10.1002/bies.20761 18478535

[B7] Boyd P. W. Jickells T. Law C. S. Blain S. Boyle E. A. Buesseler K. O. (2007). Mesoscale iron enrichment experiments 1993-2005: synthesis and future directions. *Science* 315 612–617. 10.1126/science.1131669 17272712

[B8] Caisova L. Marin B. Melkonian M. (2011). A close-up view on ITS2 evolution and speciation - a case study in the Ulvophyceae (Chlorophyta, Viridiplantae). *BMC Evol. Biol.* 11:262. 10.1186/1471-2148-11-262 21933414 PMC3225284

[B9] Chappell P. D. Whitney L. P. Haddock T. L. Menden-Deuer S. Roy E. G. Wells M. L. (2013). *Thalassiosira* spp. community composition shifts in response to chemical and physical forcing in the northeast Pacific Ocean. *Front. Microbiol.* 4:273. 10.3389/fmicb.2013.00273 24065961 PMC3779818

[B10] Chisholm S. W. Falkowski P. G. Cullen J. J. (2001). Oceans. Dis-crediting ocean fertilization. *Science* 294 309–310. 10.1126/science.1065349 11598285

[B11] Coleman A. W. (2003). ITS2 is a double-edged tool for eukaryote evolutionary comparisons. *Trends Genet.* 19 370–375. 12850441 10.1016/S0168-9525(03)00118-5

[B12] Coleman A. W. (2009). Is there a molecular key to the level of “biological species” in eukaryotes? A DNA guide. *Mol. Phylogenet. Evol.* 50 197–203. 10.1016/J.YMPEV.2008.10.008 18992828

[B13] Diner R. E. Noddings C. M. Lian N. C. Kang A. K. McQuaid J. B. Jablanovic J. (2017). Diatom centromeres suggest a mechanism for nuclear DNA acquisition. *Proc. Natl. Acad. Sci. U.S.A.* 114 E6015–E6024. 10.1073/pnas.1700764114 28673987 PMC5530661

[B14] Field C. B. Behrenfeld M. J. Randerson J. T. Falkowski P. (1998). Primary production of the biosphere: integrating terrestrial and oceanic components. *Science* 281 237–240. 10.1126/SCIENCE.281.5374.237 9657713

[B15] Fujita K. Ehira S. Tanaka K. Asai K. Ohta N. (2008). Molecular phylogeny and evolution of the plastid and nuclear encoded cbbX genes in the unicellular red alga *Cyanidioschyzon merolae*. *Genes Genet. Syst.* 83 127–133. 18506096 10.1266/ggs.83.127

[B16] Garg S. G. Gould S. B. (2016). The role of charge in protein targeting evolution. *Trends Cell Biol.* 26 894–905. 10.1016/j.tcb.2016.07.001 27524662

[B17] Gould S. B. Waller R. F. McFadden G. I. (2008). Plastid evolution. *Annu. Rev. Plant Biol.* 59 491–517. 10.1146/annurev.arplant.59.032607.092915 18315522

[B18] Groussman R. D. Parker M. S. Armbrust E. V. (2015). Diversity and evolutionary history of iron metabolism genes in diatoms. *PLoS One* 10:e0129081. 10.1371/journal.pone.0129081 26052941 PMC4460010

[B19] Gueneau P. Morel F. LaRoche J. Erdner D. (1998). The *petF* region of the chloroplast genome from the diatom *Thalassiosira weissflogii*: sequence, organization and phylogeny. *Eur. J. Phycol.* 33 203–211. 10.1017/s096702629800170x

[B20] Guillard R. R. L. (1975). “Culture of phytoplankton for feeding marine invertebrates,” in *Culture of Marine Invertebrate Animals*, eds Smith W. L. Chanley M. H., (Boston, MA: Springer), 29–60. 10.1007/978-1-4615-8714-9_3

[B21] Guillard R. R. L. Ryther J. H. (1962). Studies of marine planktonic diatoms. I. Cyclotella nana Hustedt, and Detonula confervacea (cleve) Gran. *Can. J. Microbiol.* 8 229–239. 13902807 10.1139/m62-029

[B22] Hanke G. Mulo P. (2013). Plant type ferredoxins and ferredoxin-dependent metabolism. *Plant Cell Environ.* 36 1071–1084. 10.1111/pce.12046 23190083

[B23] Huang C. Y. Ayliffe M. A. Timmis J. N. (2003). Direct measurement of the transfer rate of chloroplast DNA into the nucleus. *Nature* 422 72–76. 10.1038/nature01435 12594458

[B24] Huang X. Madan A. (1999). CAP3: a DNA sequence assembly program. *Genome Res.* 9 868–877. 10508846 10.1101/gr.9.9.868 PMC310812

[B25] Jiroutova K. Koreny L. Bowler C. Obornik M. (2010). A gene in the process of endosymbiotic transfer. *PLoS One* 5:e13234. 10.1371/journal.pone.0013234 20949086 PMC2950852

[B26] Kaczmarska I. Beaton M. Benoit A. C. Medlin L. K. (2006). Molecular phylogeny of selected members of the order thalassiosirales (Bacillariophyta) and evolution of the fultoportula1. *J. Phycol.* 42 121–138. 10.1111/j.1529-8817.2006.00161.x 27041440

[B27] Kaeriyama H. Katsuki E. Otsubo M. Yamada M. Ichimi K. Tada K. (2011). Effects of temperature and irradiance on growth of strains belonging to seven *Skeletonema* species isolated from Dokai Bay, southern Japan. *Eur. J. Phycol.* 46 113–124. 10.1080/09670262.2011.565128

[B28] Katoh K. Standley D. M. (2013). MAFFT multiple sequence alignment software version 7: improvements in performance and usability. *Mol. Biol. Evol.* 30 772–780. 10.1093/molbev/mst010 23329690 PMC3603318

[B29] Keeling P. J. Burki F. Wilcox H. M. Allam B. Allen E. E. Amaral-Zettler L. A. (2014). The marine microbial eukaryote transcriptome sequencing project (MMETSP): illuminating the functional diversity of eukaryotic life in the oceans through transcriptome sequencing. *PLoS Biol.* 12:e1001889. 10.1371/journal.pbio.1001889 24959919 PMC4068987

[B30] Kester D. R. Duedall I. W. Connors D. N. Pytkowic R. M. (1967). Preparation of artificial seawater. *Limnol. Oceanogr.* 12 176–179.

[B31] Kleffmann T. Russenberger D. von Zychlinski A. Christopher W. Sjölander K. Gruissem W. (2004). The *Arabidopsis thaliana* chloroplast proteome reveals pathway abundance and novel protein functions. *Curr. Biol.* 14 354–362. 10.1016/j.cub.2004.02.039 15028209

[B32] Kleine T. Maier U. G. Leister D. (2009). DNA transfer from organelles to the nucleus: the idiosyncratic genetics of endosymbiosis. *Annu. Rev. Plant Biol.* 60 115–138. 10.1146/annurev.arplant.043008.092119 19014347

[B33] Krasovec M. Sanchez-Brosseau S. Piganeau G. (2019). First estimation of the spontaneous mutation rate in diatoms. *Genome Biol. Evol.* 11 1829–1837. 10.1093/GBE/EVZ130 31218358 PMC6604790

[B34] Kumar M. Shukla P. K. (2005). Use of PCR targeting of internal transcribed spacer regions and single-stranded conformation polymorphism analysis of sequence variation in different regions of rRNA genes in fungi for rapid diagnosis of mycotic keratitis. *J. Clin. Microbiol.* 43 662–668. 10.1128/JCM.43.2.662-668.2005 15695661 PMC548091

[B35] LaRoche J. Murray H. Orellana M. Newton J. (1995). Flavodoxin expression as an indicator of iron limitation in marine diatoms. *J. Phycol.* 31 520–530. 10.1111/j.1529-8817.1995.tb02545.x

[B36] Leister D. (2005). Origin, evolution and genetic effects of nuclear insertions of organelle DNA. *Trends Genet.* 21 655–663. 10.1016/j.tig.2005.09.004 16216380

[B37] Lommer M. Roy A. S. Schilhabel M. Schreiber S. Rosenstiel P. LaRoche J. (2010). Recent transfer of an iron-regulated gene from the plastid to the nuclear genome in an oceanic diatom adapted to chronic iron limitation. *BMC Genomics* 11:718. 10.1186/1471-2164-11-718 21171997 PMC3022921

[B38] Lommer M. Specht M. Roy A.-S. Kraemer L. Andreson R. Gutowska M. A. (2012). Genome and low-iron response of an oceanic diatom adapted to chronic iron limitation. *Genome Biol.* 13:R66. 10.1186/gb-2012-13-7-r66 22835381 PMC3491386

[B39] Luddington I. A. Kaczmarska I. Lovejoy C. (2012). Distance and character-based evaluation of the V4 region of the 18S rRNA gene for the identification of diatoms (Bacillariophyceae). *PLoS One* 7:e45664. 10.1371/journal.pone.0045664 23029169 PMC3448646

[B40] Maldonado M. T. Price N. M. (1996). Influence of N substrate on Fe requirements of marine centric diatoms. *Mar. Ecol. Prog. Ser.* 141 161–172. 10.3354/meps141161

[B41] Martin W. F. Rujan T. Richly E. Hansen A. Cornelsen S. Lins T. (2002). Evolutionary analysis of *Arabidopsis*, cyanobacterial, and chloroplast genomes reveals plastid phylogeny and thousands of cyanobacterial genes in the nucleus. *Proc. Natl. Acad. Sci. U.S.A.* 99 12246–12251. 10.1073/pnas.182432999 12218172 PMC129430

[B42] Martin W. F. Stoebe B. Goremykin V. Hansmann S. Hasegawa M. Kowallik K. V. (1998). Gene transfer to the nucleus and the evolution of chloroplasts. *Nature* 393 162–165. 10.1038/30234 11560168

[B43] McKay R. M. L. Geider R. J. LaRoche J. (1997). Physiological and biochemical response of the photosynthetic apparatus of two marine diatoms to Fe stress. *Plant Physiol.* 114 615–622. 12223732 10.1104/pp.114.2.615 PMC158344

[B44] McKay R. M. L. LaRoche J. Yakunin A. F. Durnford D. G. Geider R. J. (1999). Accumulation of ferredoxin and flavodoxin in a marine diatom in response to Fe. *J. Phycol.* 35 510–519. 10.1046/j.1529-8817.1999.3530510.x

[B45] McQuaid J. B. Kustka A. B. Oborník M. Horák A. McCrow J. P. Karas B. J. (2018). Carbonate-sensitive phytotransferrin controls high-affinity iron uptake in diatoms. *Nature* 555 534–537. 10.1038/nature25982 29539640

[B46] Medlin L. K. Williams D. M. Sims P. A. (1993). The evolution of the diatoms origin of the group and assessment of the monophyly of its major divisions. *Eur. J. Phycol.* 28 261–275. 10.1080/09670269300650381

[B47] Moniz M. B. J. Kaczmarska I. (2009). Barcoding diatoms: is there a good marker? *Mol. Ecol. Resour*. 9(Suppl. 1), 65–74. 10.1111/j.1755-0998.2009.02633.x 21564966

[B48] Moore J. K. Doney S. C. Lindsay K. (2004). Upper ocean ecosystem dynamics and iron cycling in a global three-dimensional model. *Glob. Biogeochem. Cycles* 18:GB4028 10.1029/2004GB002220

[B49] Nelson D. M. Tréguer P. Brzezinski M. A. Leynaert A. Quéguiner B. (1995). Production and dissolution of biogenic silica in the ocean: revised global estimates, comparison with regional data and relationship to biogenic sedimentation. *Glob. Biogeochem. Cycles* 9 359–372. 10.1029/95GB01070

[B50] Nguyen L.-T. Schmidt H. A. von Haeseler A. Minh B. Q. (2015). IQ-TREE: a fast and effective stochastic algorithm for estimating maximum-likelihood phylogenies. *Mol. Biol. Evol.* 32 268–274. 10.1093/molbev/msu300 25371430 PMC4271533

[B51] OceanStudiesBoard, (2010). *Ocean Acidification: A National Strategy to Meet the Challenges of a Changing Ocean.* Washington, DC: The National Academies Press.

[B52] O’Leary N. A. Wright M. W. Brister J. R. Ciufo S. Haddad D. McVeigh R. (2016). Reference sequence (RefSeq) database at NCBI: current status, taxonomic expansion, and functional annotation. *Nucleic Acids Res.* 44 D733–D745. 10.1093/nar/gkv1189 26553804 PMC4702849

[B53] Oudot-Le Secq M.-P. Grimwood J. Shapiro H. Armbrust E. V. Bowler C. Green B. R. (2007). Chloroplast genomes of the diatoms *Phaeodactylum tricornutum* and *Thalassiosira pseudonana*: comparison with other plastid genomes of the red lineage. *Mol. Genet. Genomics* 277 427–439. 17252281 10.1007/s00438-006-0199-4

[B54] Peers G. Price N. M. (2006). Copper-containing plastocyanin used for electron transport by an oceanic diatom. *Nature* 441 341–344. 10.1038/nature04630 16572122

[B55] Pelham R. J. Rodgers L. Hall I. Lucito R. Nguyen K. C. Q. Navin N. (2006). Identification of alterations in DNA copy number in host stromal cells during tumor progression. *Proc. Natl. Acad. Sci. U.S.A.* 103 19848–19853. 10.1073/pnas.0609635104 17167050 PMC1698871

[B56] Pitti R. M. Marsters S. A. Lawrence D. A. Roy M. Kischkel F. C. Dowd P. (1998). Genomic amplification of a decoy receptor for Fas ligand in lung and colon cancer. *Nature* 396 699–703. 10.1038/25387 9872321

[B57] Pruitt K. D. Tatusova T. Brown G. R. Maglott D. R. (2012). NCBI reference sequences (RefSeq): current status, new features and genome annotation policy. *Nucleic Acids Res.* 40 D130–D135. 10.1093/nar/gkr1079 22121212 PMC3245008

[B58] Quast C. Pruesse E. Yilmaz P. Gerken J. Schweer T. Yarza P. (2013). The SILVA ribosomal RNA gene database project: improved data processing and web-based tools. *Nucleic Acids Res.* 41 D590–D596. 10.1093/nar/gks1219 23193283 PMC3531112

[B59] Rice P. Longden I. Bleasby A. (2000). EMBOSS: the European molecular biology open software suite. *Trends Genet.* 16 276–277. 10827456 10.1016/s0168-9525(00)02024-2

[B60] Richly E. Leister D. (2004). NUPTs in sequenced eukaryotes and their genomic organization in relation to NUMTs. *Mol. Biol. Evol.* 21 1972–1980. 10.1093/molbev/msh210 15254258

[B61] Round F. E. (1990). Diatom communities - their response to changes in acidity. *Philos. Trans. R. Soc. Lond. Ser. B Biol. Sci.* 327 243–249. 10.1098/rstb.1990.0059

[B62] Ruck E. C. Nakov T. Jansen R. K. Theriot E. C. Alverson A. J. (2014). Serial gene losses and foreign DNA underlie size and sequence variation in the plastid genomes of diatoms. *Genome Biol. Evol.* 6 644–654. 10.1093/gbe/evu039 24567305 PMC3971590

[B63] Sabir J. S. M. Yu M. Ashworth M. P. Baeshen N. A. Baeshen M. N. Bahieldin A. (2014). Conserved gene order and expanded inverted repeats characterize plastid genomes of Thalassiosirales. *PLoS One* 9:e107854. 10.1371/journal.pone.0107854 25233465 PMC4169464

[B64] Senapin S. Phiwsaiya K. Kiatmetha P. Withyachumnarnkul B. (2011). Development of primers and a procedure for specific identification of the diatom *Thalassiosira weissflogii*. *Aquac. Int.* 19 693–704. 10.1007/s10499-010-9384-x

[B65] Sheppard A. E. Timmis J. N. (2009). Instability of plastid DNA in the nuclear genome. *PLoS Genet.* 5:e1000323. 10.1371/journal.pgen.1000323 19119415 PMC2602989

[B66] Smith D. R. (2015). Mutation rates in plastid genomes: they are lower than you might think. *Genome Biol. Evol.* 7 1227–1234. 10.1093/gbe/evv069 25869380 PMC4453064

[B67] Sorhannus U. Ortiz J. D. Wolf M. Fox M. G. (2010). Microevolution and speciation in *Thalassiosira weissflogii* (Bacillariophyta). *Protist* 161 237–249. 10.1016/j.protis.2009.10.003 20018562

[B68] Stegemann S. Hartmann S. Ruf S. Bock R. (2003). High-frequency gene transfer from the chloroplast genome to the nucleus. *Proc. Natl. Acad. Sci. U.S.A.* 100 8828–8833. 10.1073/pnas.1430924100 12817081 PMC166398

[B69] Strzepek R. F. Harrison P. J. (2004). Photosynthetic architecture differs in coastal and oceanic diatoms. *Nature* 431 689–692. 10.1038/nature02954 15470428

[B70] Sunda W. G. Huntsman S. A. (1995). Iron uptake and growth limitation in oceanic and coastal phytoplankton. *Mar. Chem.* 50 189–206. 10.1016/0304-4203(95)00035-P

[B71] Sunda W. G. Swift D. G. Huntsman S. A. (1991). Low iron requirement for growth in oceanic phytoplankton. *Nature* 351 55–57. 10.1038/351055a0

[B72] Theriot E. C. Ashworth M. P. Nakov T. Ruck E. Jansen R. K. (2015). Dissecting signal and noise in diatom chloroplast protein encoding genes with phylogenetic information profiling. *Mol. Phylogenet. Evol.* 89 28–36. 10.1016/j.ympev.2015.03.012 25848969

[B73] Thorsness P. E. Fox T. D. (1990). Escape of DNA from mitochondria to the nucleus in *Saccharomyces cerevisiae*. *Nature* 346 376–379. 10.1038/346376a0 2165219

[B74] Timmis J. N. Ayliffe M. A. Huang C. Y. Martin W. F. (2004). Endosymbiotic gene transfer: organelle genomes forge eukaryotic chromosomes. *Nat. Rev. Genet.* 5 123–135. 10.1038/nrg1271 14735123

[B75] von Dassow P. Petersen T. W. Chepurnov V. A. Virginia Armbrust E. (2008). Inter- and intraspecific relationships between nuclear DNA content and cell size in selected members of the centric diatom genus *Thalassiosira* (Bacillariophyceae). *J. Phycol.* 44 335–349. 10.1111/j.1529-8817.2008.00476.x 27041190

[B76] Wang D. Lloyd A. H. Timmis J. N. (2012). Environmental stress increases the entry of cytoplasmic organellar DNA into the nucleus in plants. *Proc. Natl. Acad. Sci. U.S.A.* 109 2444–2448. 10.1073/pnas.1117890109 22308419 PMC3289323

[B77] Wang D. Timmis J. N. (2013). Cytoplasmic organelle DNA preferentially inserts into open chromatin. *Genome Biol. Evol.* 5 1060–1064. 10.1093/gbe/evt070 23661564 PMC3698918

[B78] Whitney L. P. Lins J. J. Hughes M. P. Wells M. L. Dreux Chappell P. Jenkins B. D. (2011). Characterization of putative iron responsive genes as species-specific indicators of iron stress in thalassiosiroid diatoms. *Front. Microbiol.* 2:234. 10.3389/fmicb.2011.00234 22275908 PMC3223615

[B79] Whittaker K. A. Rignanese D. R. Olson R. J. Rynearson T. A. (2012). Molecular subdivision of the marine diatom *Thalassiosira rotula* in relation to geographic distribution, genome size, and physiology. *BMC Evol. Biol.* 12:209. 10.1186/1471-2148-12-209 23102148 PMC3544637

[B80] Wolfe K. H. Li W. H. Sharp P. M. (1987). Rates of nucleotide substitution vary greatly among plant mitochondrial, chloroplast, and nuclear DNAs. *Proc. Natl. Acad. Sci. U.S.A.* 84 9054–9058. 3480529 10.1073/pnas.84.24.9054 PMC299690

[B81] Woodson J. D. Chory J. (2008). Coordination of gene expression between organellar and nuclear genomes. *Nat. Rev. Genet.* 9 383–395. 10.1038/nrg2348 18368053 PMC4854206

[B82] Yan T. Zhou M. Qian P. (2002). Combined effects of temperature, irradiance and salinity on growth of diatom *Skeletonema costatum*. *Chin. J. Oceanol. Limnol.* 20 237–243.

[B83] Zimorski V. Ku C. Martin W. F. Gould S. B. (2014). Endosymbiotic theory for organelle origins. *Curr. Opin. Microbiol.* 22 38–48. 10.1016/j.mib.2014.09.008 25306530

