# Supplementary material for: The Transfer of the Ferredoxin Gene From the Chloroplast to the Nuclear Genome Is Ancient Within the Paraphyletic Genus Thalassiosira
Source: Front Microbiol. 2020 Oct 2;11:523689. doi: 10.3389/fmicb.2020.523689 (PMC7566914; doi:10.3389/fmicb.2020.523689)
Supplement: Supplementary Figure S1 — Peptide homologs showing target peptide at the N-terminus typically found for plastid-targeted proteins encoded on the nucleus. A label indicates if the target peptide is suspected to be present; for sequences 8 & 9 information about the targeting peptide is missing. If protein sequences from public databases were modified for the alignment, the legend exhibits information on the details (See ‘Note:’). [file Image_1.pdf]

No target peptide (sequences 1–9); Target peptide (sequences 10–19)

```
    1
1   ---------M S--------- ---------- ---------- --------IY SVVLKSEEHD IDTTVDCNDD VFILDAAEEN
2   ---------M A--------- ---------- ---------- --------TY KVTLLSEEHD IDTTLDCPDD AFILDVAEEQ
3   ---------M A--------- ---------- ---------- --------SY NVTLVSEEHD IDQTIECQDS EYILDAAEEK
4   ---------M A--------- ---------- ---------- --------TY KVTLISEEHD INTTIECNDD VFVLDAAEEA
5   ---------M A--------- ---------- ---------- --------TY KVTLISEEHD INATIDCNDD VFVLDAAEEA
6   ---------M A--------- ---------- ---------- --------TY KVTLISEEHD INTTIDCNDD VFVLDAAEEA
7   ---------M A--------- ---------- ---------- --------TY KVTLISEEHD INTTIDCNDD VFVLDAAEEA
8   ---------- ---------- ---------- ---------- ---------- ---------- ---------- ---VDAAEEA
9   ---------- ---------- ---------- ---------- ---------- ---------- --------DD VFIVDAAEEA
10  ---------M KLAIIAACVA SAAAFAP--- -MA-P-V--- --RQATSLDY SVKVFNEEEG IDATFECADD VFIVDAAEEE
11  ---------M KLAIAAILVA SAAAFAP--- -SA-PVV--- --SRTTSLGY SIHVVNEDEG IDATFECADD SFLVDAAEEE
12  ---------M KLAIAATLVA SAAAFAP--- -SA-P-VIS- -RVSSTSLDY SVHVFNEEEG IDATFECADD ATIVDAAEDE
13  ---------M KLTIAAVLCA SAAAFAP--- -SA-P-VVS- -RVASTSLGW TVHVYNEEEE IDATFDCEEG TYIVDAAEEA
14  ---------M KLAIAAILVA SAAAFAP--- -SA-PVV--- --SRTTSLGY SIHVFNEDEG IDSTFDCADD SFLVDAAEEA
15  ---------M KLAIATLLVA SAAAFAP--- -SA-PVV--- --TRTTSLGY SVKVVNEDEG IDATFECADD SFIVDAAEEA
16  MFSKTL---I A----AVLAV SAQGFAPTPR SAV-T-V--- --RQQTSLGY AVKLISEEHE IDATVEVADD VYIVDAAEEE
17  MFTKALL-VL A------AAS VAQGFSP--- -VARPMT--- --RSSTSLGY SVQLVSEEEG LDETIECADD MFIVDAAEDA
18  ---------M KLAIIAACVA SAAAFAP--- -MA-P-V--- --RQATSLDY SVKVFNEEEG IDATFECADD VFIVDAAEEE
19  MFSKTLFALL A--------V SVEGFAP--- -IV-P-VSTV VGRQSTSLGF TVKLISEEHG MNTSIECPDD EMIVDAAERA
                              Target peptide

   81
1   GIDLPYSCRA GACSTCAGKV VEGTIDQSEQ TFLDDDQVGA GFVLTCIAYP KSDCTILVHQ EDELY-
2   GIELPYSCRA GACSTCAGKV VEGTIDQSEQ SFLDEDQVAA GFVLTCVMYP TSDCKILVHQ EDELY-
3   GIDLPYSCKS GACSSCAGKI VEGEIDQSEQ SFLEDDQVDQ GFVLTCVAYP KSDCKILVHQ EDELY-
4   GIDLPYSCRA GACSTCAGKV TEGSIDQSEQ TFLDDDQMGA GFVLTCIAYP KSDCTILVHQ EDELY-
5   GIDLPYSCRA GACSTCAGKV SDGTIDQSEQ TFLDDDQMGA GFVLTCIAYP KSDCTILVHQ EDELY-
6   GIDLPYSCRA GACSTCAGKV TEGTIDQSEQ TFLDDDQMGA GFVLTCIAYP KSDCTILVHQ EDELY-
7   GIDLPYSCRA GACSTCAGKV TEGTIDQSEQ TFLDDDQMGA GFVLTCIAYP KSDCTILVHQ EDELY-
8   GIDLPYSCRA GACSTCAGKI TEGEVDQSEQ TFLDDDQMAD GFVLTCVAYP KGDCTIAVHM EDELF-
9   GIELPYSCRA GACSTCAGKV LEGEVDQSEQ TFLDDDQMDD GWVLTCVAYP KGDCTIAVHM EDELF-
10  GVDLPYSCRA GACSTCTGKV ISGEVDQSEQ TFLDDDQMAD GYVLTCVAYP KSDCEIQVHM EDDLF-
11  GIDLPYSCRA GACSTCTGKM ISGEVDQSEQ TFLDDDQMGD GFVLTCVAYP KSDCEIQVHM EDELF-
12  GIDLPYSCRA GACSTCAGKI ISGTVNQSEQ NFLEDEQIDD GFVLTCVAFP TSDCEIAVHM EEDLF-
13  GVDLPYSCRA GACSTCVGKI EGGEVNQEEQ SFLDEDQMEE GWALTCVAFP TADCEIKVHM EEDLFG
14  GIXLPYSCRA GACSTCTGKM ISGEVDQSEQ TFLDDDQMGD GFVLTCVAYP KSDCEIQVHM EDDLF-
15  GIDLPYSCRA GACSTCTGKI ISGTVDQSEQ TFLDDDQMGE GFVLTCVAYP TSDCEIQVHM EDELF-
16  GVDLPYSCKA GACSTCAGKV LEGTVDQSEQ TFLEDEQMDE GWVLTCVALP TSDCTIQVHM EDDLF-
17  GVELPYSCRA GACSTCTGKV LDGSVDQSEQ TFLDDDQMAD GYVLTCVAYP TSDCKIEVHM EDDLF-
18  GVDLPYSCRA GACSTCTGKV ISGEVDQSEQ TFLDDDQMAD GYVLTCVAYP KSDCEIQVHM EDDLF-
19  GLDLPYSCRA GACSTCTGKV IEGTVDQSDQ TFLEDEQMNE GWVLTCVAYP TSDCTIQTHM EDDLV-
```

Sequence legend:

1. Chaetoceros_simplex (YP_009092887.1)
2. Coscinodiscus_radiatus (YP_009028889.1)
3. Rhizosolenia_imbricata (YP_009093033.1)
4. Roundia_cardiophora (YP_009093137.1)
5. Thalassiosira_pseudonana (YP_874492.1)
6. Thalassiosira_weissflogii (YP_009093409.1)
7. Thalassiosira_weissflogii_Strain_CCMP1010_MMETSP1408 (CAMPEP_0203187078; Note: Removed "Q" from protein start and "X" from protein end)
8. Thalassiosira_sp_Strain_NH16_MMETSP1071 (CAMPEP_0181094700; Note: Protein start incomplete, Removed "X" from protein start)
9. Thalassiosira_punctigera_Strain_Tpunct2005C2_MMETSP1067 (CAMPEP_0172528362; Note: Protein start incomplete)
10. Thalassiosira_oceanica (EJK54785.1)
11. Skeletonema_dohrnii_Strain_SkelB_MMETSP0562 (CAMPEP_0195172450)
12. Skeletonema_grethea_Strain_CCMP_1804_MMETSP0578 (CAMPEP_0201696492; Note: Removed "XFKCLKVCNYLHTTTIKM" at protein start)
13. Skeletonema_japonicum_Strain_CCMP2506_MMETSP0593 (CAMPEP_0201726936; Note: Removed "XIIHTYPSNTIK" at protein start)
14. Skeletonema_marinoi_Strain_SM1012Hels-07_MMETSP0319 (CAMPEP_0115919778)
15. Skeletonema_menzelii_Strain_CCMP793_MMETSP0603 (CAMPEP_0183648216)
16. Thalassiosira_gravida_Strain_GMp14c1_MMETSP0492 (CAMPEP_0201608532)
17. Thalassiosira_miniscula_Strain_CCMP1093_MMETSP0737 (CAMPEP_0183720344)
18. Thalassiosira_oceanica_Strain_CCMP1005_MMETSP0970 (CAMPEP_0202142184)
19. Thalassiosira_rotula_Strain_GSO102_MMETSP0913 (CAMPEP_0196233982; Note: Removed "XSLAAIRLLPPNNI" at protein start)
