# Supplementary figures and images for: The Transfer of the Ferredoxin Gene From the Chloroplast to the Nuclear Genome Is Ancient Within the Paraphyletic Genus Thalassiosira
Source: Front Microbiol. 2020 Oct 2;11:523689. doi: 10.3389/fmicb.2020.523689 (PMC7566914; doi:10.3389/fmicb.2020.523689)

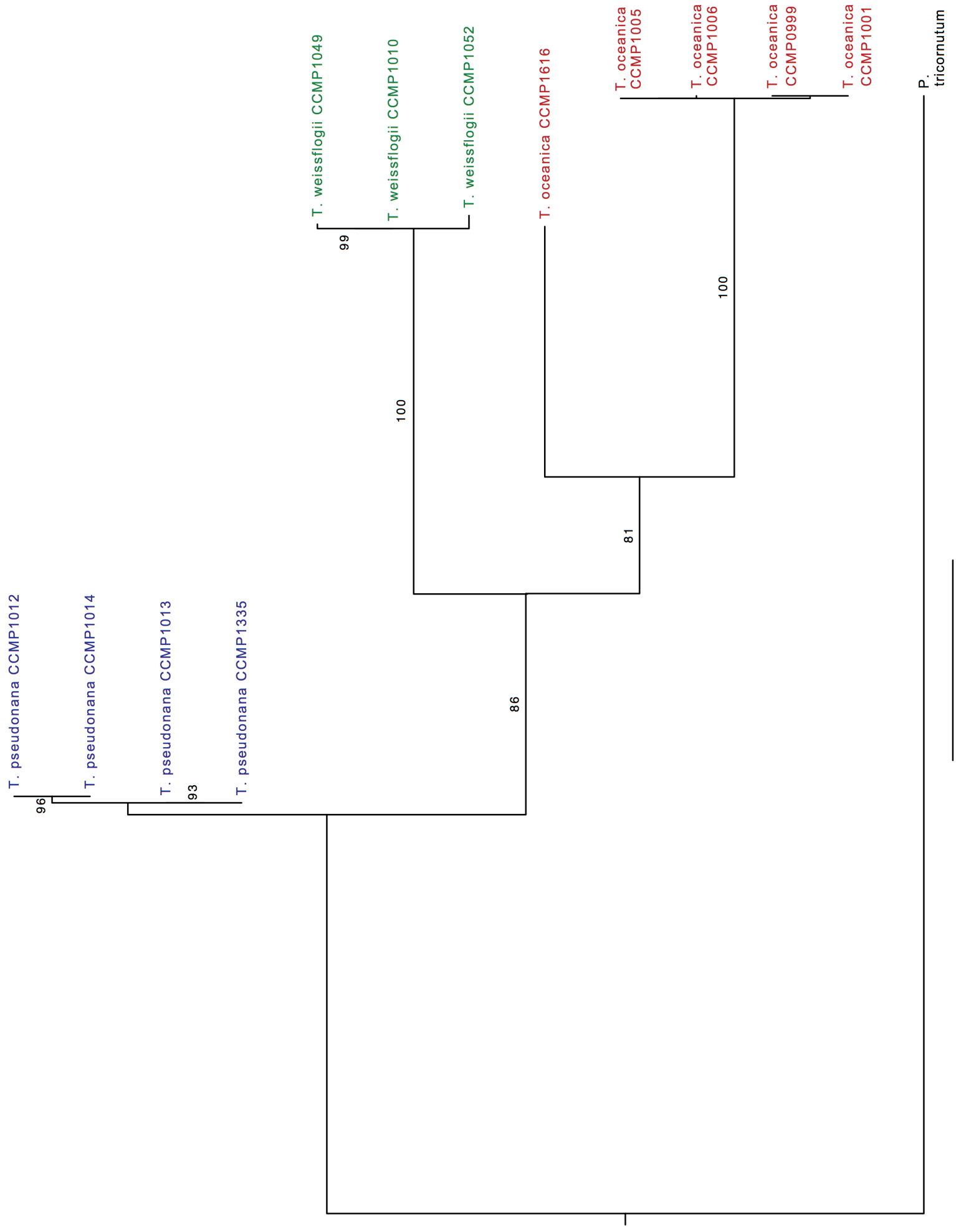

Supplement: Supplementary Figure S2 — ITS2 phylogenetic tree of experimentally cultured Thalassiosira (colored) with Phaeodactylum tricornutum as outgroup root; where only bootstraps values above 70 are presented. [file Image_2.pdf]

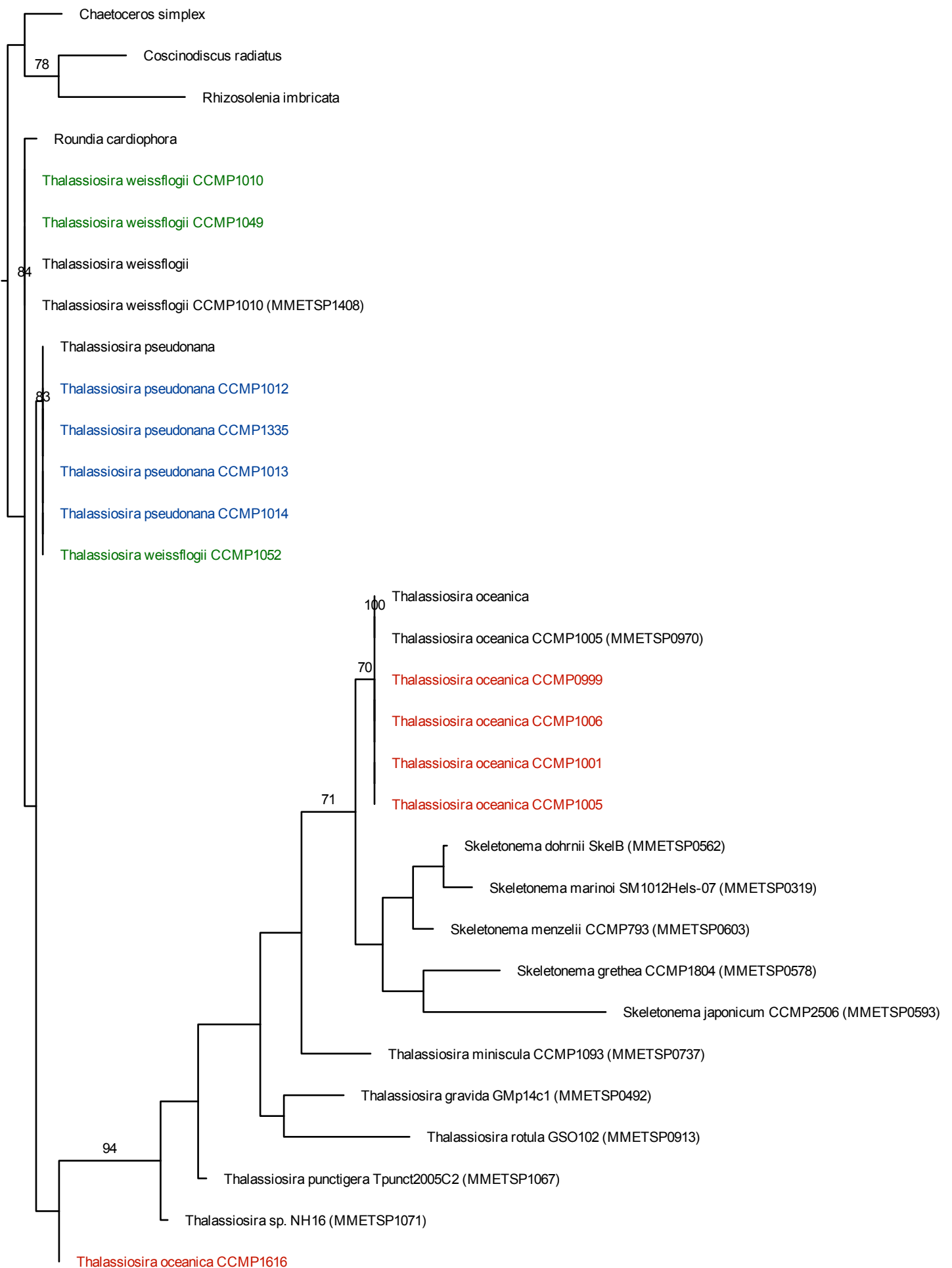

0.2

Supplement: Supplementary Figure S3 — Phylogenetic tree of ferredoxin (PetF) protein sequences without target peptides where experimentally cultured Thalassiosira strains are colored. Parametric bootstraps values above 70 are presented at the branches. The outgroup root clade contains Rhizosolenia imbricata, Coscinodiscus radiatus, and Chaetoceros simplex. For details on the sequences added from public databases see Supplementary Figure S1. [file Image_3.pdf]
