# Supplementary material for: The Transfer of the Ferredoxin Gene From the Chloroplast to the Nuclear Genome Is Ancient Within the Paraphyletic Genus Thalassiosira
Source: Front Microbiol. 2020 Oct 2;11:523689. doi: 10.3389/fmicb.2020.523689 (PMC7566914; doi:10.3389/fmicb.2020.523689)
Supplement: Supplementary Table S2 — Oligonucleotides primers used for qPCR analysis 1 (gray shading) and for Sanger sequencing. [file Table_2.pdf]

Table S2. Oligonucleotides primers used for qPCR analysis (grey shading) and for Sanger sequencing.

**Species**	**Genes**	**Forward primers (5’→ 3’)**	**Reverse primers (5’→ 3’)**
Thalassiosira sp.	*18S*	GGATTTCTGGCAGGAGCGA	GCCCGACAACTTAATGCCAA
T. oceanica	*petF*	GAGGTTGACCAGTCCGAGCA	GGATCTCGCAGTCCGACTTG
T. pseudonana/weissflogii	*petF*	CGTGCTGGTGCTTGTTCTACAT	CAAAACCAGCACCCATTTGAT
T. oceanica	*tef1a*	GTCCGTCTCCCCATCTCCA	CAACAACATCTCCAGGGACGA
T. pseudonana	*tef1a*	CCACTACACCATTGTCGATGCT	GGCAGGCACAAGAAGAAGACC
T. weissflogii	*tef1a*	CTTCCGGTTCGTCTTCCCAT	CATCTCCCGGACGAAGAGTTC
T. oceanica	*rbcS*	TAGAGTGGACAGATGATCCGCA	AATTCAAACATTACTGTCGCAGGA
T. pseudonana	*rbcS*	TAGAATGGACAGATGATCCACA	AATTCGAACATTACTGTTGCAGGA
T. weissflogii	*rbcS*	TAGAGTGGACAGATGATCCACA	AATTCGAACATTACTGTCGCAGGA
T. oceanica	*petF*	TTGACCTTCCTTACTCGTGCC	CAGTCCGACTTGGGGTAAGC
	ITS1-5.8S-ITS2	TCCGTAGGTGAACCTGCGG	GCTTAAATTCGGCGGGTAGTCT
T. pseudonana and *T. weissflogii*	*petF*	GCAGCAGAAGAAGCAGGAATTG	CAGTCTGATTTTGGATATGC
	ITS1-5.*8S*-ITS2	TCCGTAGGTGAACCTGCGG	TCCTCCGCTTATTAATATG
Thalassiosira spp.	*18S*	GGATTTCTGGCAGGAGCGA	TTCACCTACGGAAACCTTGTTA
